# Renal tubular peroxisomes are dispensable for normal kidney function

**DOI:** 10.1172/jci.insight.155836

**Published:** 2022-02-22

**Authors:** Camille Ansermet, Gabriel Centeno, Sylvain Pradervand, Dusan Harmacek, Andy Garcia, Jean Daraspe, Sai Kocherlakota, Myriam Baes, Yohan Bignon, Dmitri Firsov

**Affiliations:** 1 Department of Biomedical Sciences,; 2 Genomic Technologies Facility, and; 3 Electron Microscopy Facility, University of Lausanne, Lausanne, Switzerland.; 4 Department for Pharmaceutical and Pharmacological Sciences, University of Leuven, Leuven, Belgium.

**Keywords:** Nephrology, Genetic diseases

## Abstract

Peroxisomes are specialized cellular organelles involved in a variety of metabolic processes. In humans, mutations leading to complete loss of peroxisomes cause multiorgan failure (Zellweger’s spectrum disorders, ZSD), including renal impairment. However, the (patho)physiological role of peroxisomes in the kidney remains unknown. We addressed the role of peroxisomes in renal function in mice with conditional ablation of peroxisomal biogenesis in the renal tubule (cKO mice). Functional analyses did not reveal any overt kidney phenotype in cKO mice. However, infant male cKO mice had lower body and kidney weights, and adult male cKO mice exhibited substantial reductions in kidney weight and kidney weight/body weight ratio. Stereological analysis showed an increase in mitochondria density in proximal tubule cells of cKO mice. Integrated transcriptome and metabolome analyses revealed profound reprogramming of a number of metabolic pathways, including metabolism of glutathione and biosynthesis/biotransformation of several major classes of lipids. Although this analysis suggested compensated oxidative stress, challenge with high-fat feeding did not induce significant renal impairments in cKO mice. We demonstrate that renal tubular peroxisomes are dispensable for normal renal function. Our data also suggest that renal impairments in patients with ZSD are of extrarenal origin.

## Introduction

Peroxisomes are single-membrane-bound organelles that were first found in mouse kidney by Rhodin in 1954 (1). Current estimates suggest that peroxisomes contain more than 50 enzymes involved in a variety of cellular functions, including β-oxidation of very-long-chain fatty acids (VLCFAs), long-chain fatty acids (LCFAs), and long-chain dicarboxylic acid; α- and β-oxidation of branched-chain fatty acids; oxidation of prostaglandins and leukotrienes; metabolism of amino acids; biosynthesis of ether phospholipids (including plasmalogens) and bile acids; redox homeostasis; glyoxylate detoxification; and ferroptosis. Peroxisomes are ubiquitously present in nearly all mammalian cells, with the highest abundance in kidney proximal tubule cells and in hepatocytes. In these cells, peroxisomes occupy about 3% of cell volume, but their number, size, and shape vary significantly during embryonic and postembryonic development (2); their number can grow greatly in various stress conditions (3). Although many peroxisomal enzymes have been well characterized, studies addressing their expression and functional role in the kidney are scarce. Moreover, the overall functional relevance of peroxisomes in the kidney remains largely unknown.

Evidence for a role of peroxisomes in renal (patho)physiology has mainly emerged from human genetics studies. Two types of mutations leading to human peroxisomal disorders have been identified: (i) mutations in so-called peroxin (*PEX*) genes involved in peroxisome biogenesis and (ii) mutations in specific peroxisomal enzymes. The first type of mutations lead to generalized peroxisomal dysfunction causing cerebro-hepato-renal Zellweger’s spectrum disorders (ZSD) (4). Infants with severe forms of ZSD usually die during the first year of life from multiorgan failure. Even though the presence of cortical renal cysts and/or renal oxalate stones in patients with ZSD has been well documented over years (5, 6), molecular mechanisms leading to these impairments remain so far unknown. Other possible renal abnormalities in ZSD have not been investigated because the disease is dominated by neurological complications. The impact of intermediate or milder forms of ZSD on renal function has not been systemically evaluated. Evidence linking single peroxisomal enzyme deficiency to renal pathophysiology also remains limited. Mutations in the liver peroxisomal alanine:glyoxylate aminotransferase encoded by the *AGXT* gene lead to primary hyperoxaluria type I, a disease characterized by deposition of calcium oxalate crystals in the kidney (reviewed in ref. 7). Recently, an autosomal dominant mutation leading to the renal Fanconi syndrome, or generalized dysfunction of the proximal tubule, has been identified in the peroxisomal enzyme enoyl-CoA hydratase and 3-hydroxyacyl CoA dehydrogenase (EHHADH) (8). This mutation causes mistargeting of EHHADH to mitochondria and impairment of mitochondrial function. Genetic studies performed on congenic rat strains have shown that the peroxisomal enzyme hydroxyacid oxidase 2 (HAO2) may play a role in blood pressure control (9, 10). However, whether this phenotype is due to the altered HAO2 function in the kidney or in other tissues remains unknown. In general, data from animal models with kidney-specific defect of peroxisome biogenesis or kidney-specific inactivation of a single peroxisomal enzyme are still missing. Here, we addressed the role of peroxisomes in renal function in male and female mice with conditional ablation of peroxisomal biogenesis in the renal tubule.

## Results

### Generation and basic characterization of infant male and female mice with conditional ablation of peroxisomal biogenesis in the renal tubule.

Peroxisome biogenesis in the renal tubule was disrupted through conditional inactivation of *Pex5* encoding cytosolic peroxisome targeting signal 1 (PTS1) receptor, which is essential for import of PTS1 motif-containing proteins into peroxisomes (11, 12). Conditional deletion of *Pex5* in the renal tubule was achieved using a doxycycline-inducible (DOX-inducible) system (*Pex5*^lox/lox^/*Pax8-rtTA*/LC1 mice, ref. 13). Because functional importance of peroxisomes may be age dependent (14), basic characterization of *Pex5* deficiency in the kidney was performed both in infant and in adult mice. In experiments with infant mice, excision of floxed *Pex5* allele was achieved through administration of DOX (2 mg/mL in drinking water) to pregnant females at E14 and maintained until P7. Infant mice were sacrificed at P28. Mice with *Pex5*^lox/lox^ genotype were used as controls. As shown in Supplemental Figure 1A (supplemental material available online with this article; https://doi.org/10.1172/jci.insight.155836DS1), DOX treatment resulted in near-complete excision of floxed *Pex5* allele in *Pex5*^lox/lox^/*Pax8-rtTA*/LC1 male and female infant mice. Analysis of renal sections did not reveal any overt histological abnormalities or renal stones in infant mice devoid of *Pex5* in the renal tubule (not shown). Albuminuria was absent in spot urine samples of Pex5-devoid infant mice of both sexes (Supplemental Figure 1B). Body weight (BW) and kidney weight (KW) but not KW/BW ratio were lower in male Pex5-devoid infant mice compared with littermate controls (Supplemental Figure 1C).

### Generation and basic characterization of adult male and female mice with conditional ablation of peroxisomal biogenesis in the renal tubule.

**Figure 1:**
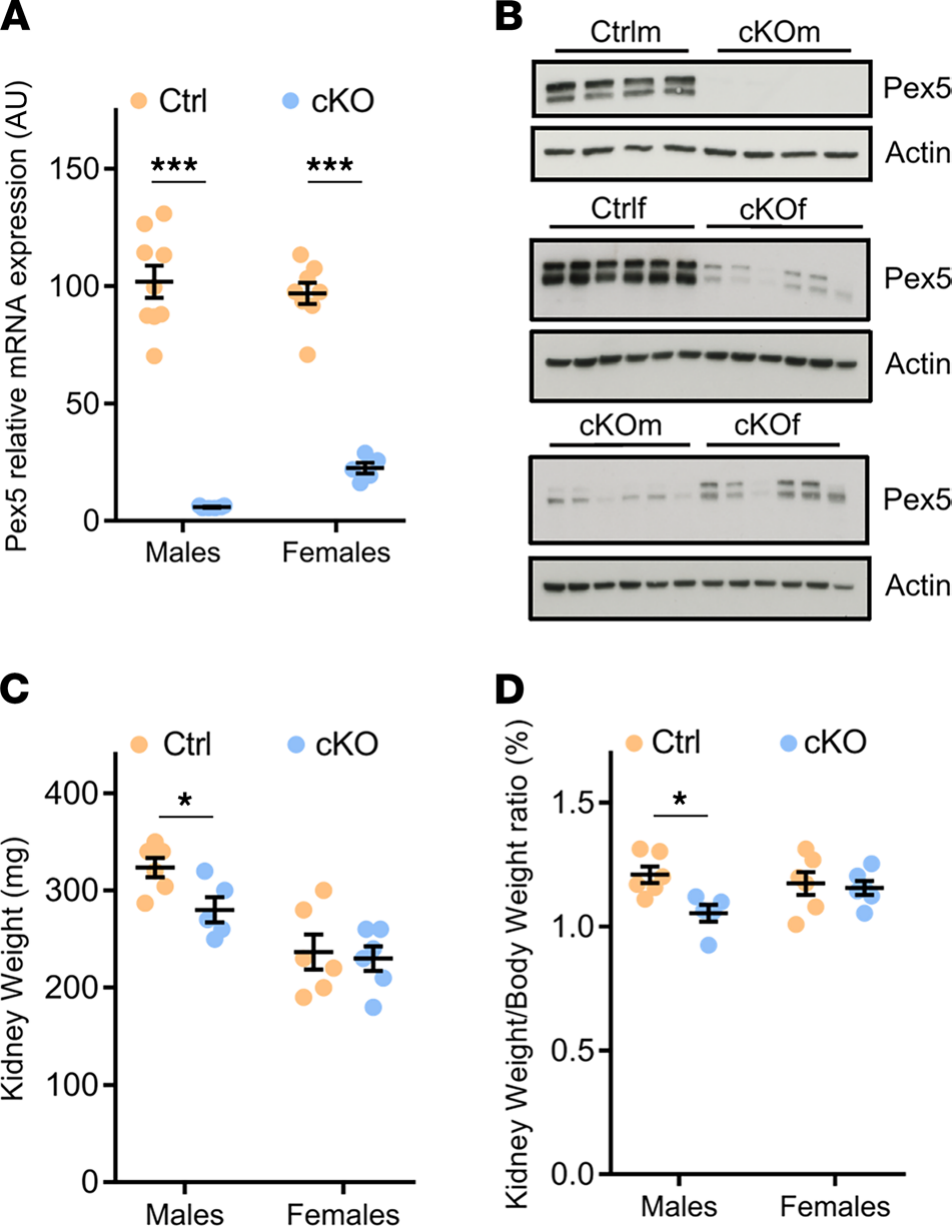
Validation of the cKO model and basic characteristics of cKOm and cKOf mice. (**A**) Relative *Pex5* mRNA expression in kidneys of Ctrlm, cKOm, Ctrlf, and cKOf mice (*n* = 5–9). One hundred percent corresponds to the mean of *Pex5* mRNA expression in kidneys of Ctrlm mice. (**B**) Western blot analysis of PEX5 protein expression in kidneys of cKOm and cKOf or of Ctrlf and cKOf mice. (**C**) Kidney weights of Ctrlm, cKOm, Ctrlf, and cKOf mice (*n* = 12–13). (**D**) Kidney weight/body weight ratio for Ctrlm, cKOm, Ctrlf, and cKOf mice (*n* = 5–6). Box and whiskers represent mean ± SEM; unpaired *t* test, ***P < 0.0001, *P < 0.05. The original full-length Western blot images for **B** are shown in Supplemental Figure 9.

The *Pex5* deletion in the renal tubule of adult mice was induced by 2-week treatment with DOX of 8-week-old *Pex5*^lox/lox^/*Pax8-rtTA*/LC1 male and female mice, hereinafter referred to as cKOm and cKOf mice, respectively (see Methods). In parallel, the same DOX treatment was provided to their littermate controls (*Pex5*^lox/lox^ mice, hereafter referred to as Ctrlm and Ctrlf mice, respectively). All experiments on adult mice were performed 4 weeks after the end of DOX treatment. As shown in Figure 1A, *Pex5* mRNA expression was significantly reduced in kidneys of both cKOm and cKOf mice. However, in cKOf mice, some residual full-length *Pex5* mRNA was also detectable, suggesting incomplete excision of floxed allele. Similarly, PEX5 protein was virtually absent in kidneys of cKOm mice but still detectable in cKOf mice, albeit at a substantially lower level. This difference was clearly visible when kidney extracts from cKOm and cKOf mice were loaded on the same SDS-PAGE gel and immunoblotted together (Figure 1B). Both cKOm and cKOf mice were viable, displayed no overt abnormalities, and had normal BW compared to control mice (Supplemental Table 1). The KW and KW/BW ratio were substantially lower in cKOm mice compared with Ctrlm mice but not in cKOf mice compared to Ctrlf mice (Figure 1, C and D, respectively). Analysis of 24-hour urine and plasma samples did not reveal an effect of genotype in mice of both sexes, except lower plasma potassium levels in cKOm mice compared with Ctrlm mice (Supplemental Table 1). Albuminuria was absent in urine of both cKOm and cKOf mice (Supplemental Figure 2A). No gross morphological changes, calcium oxalate deposits, or lipid accumulation were observed in the kidney of cKOm mice (Supplemental Figure 2, B–D, respectively).

### Electron microscopy assessment of proximal tubule cells in adult cKO mice.

**Figure 2:**
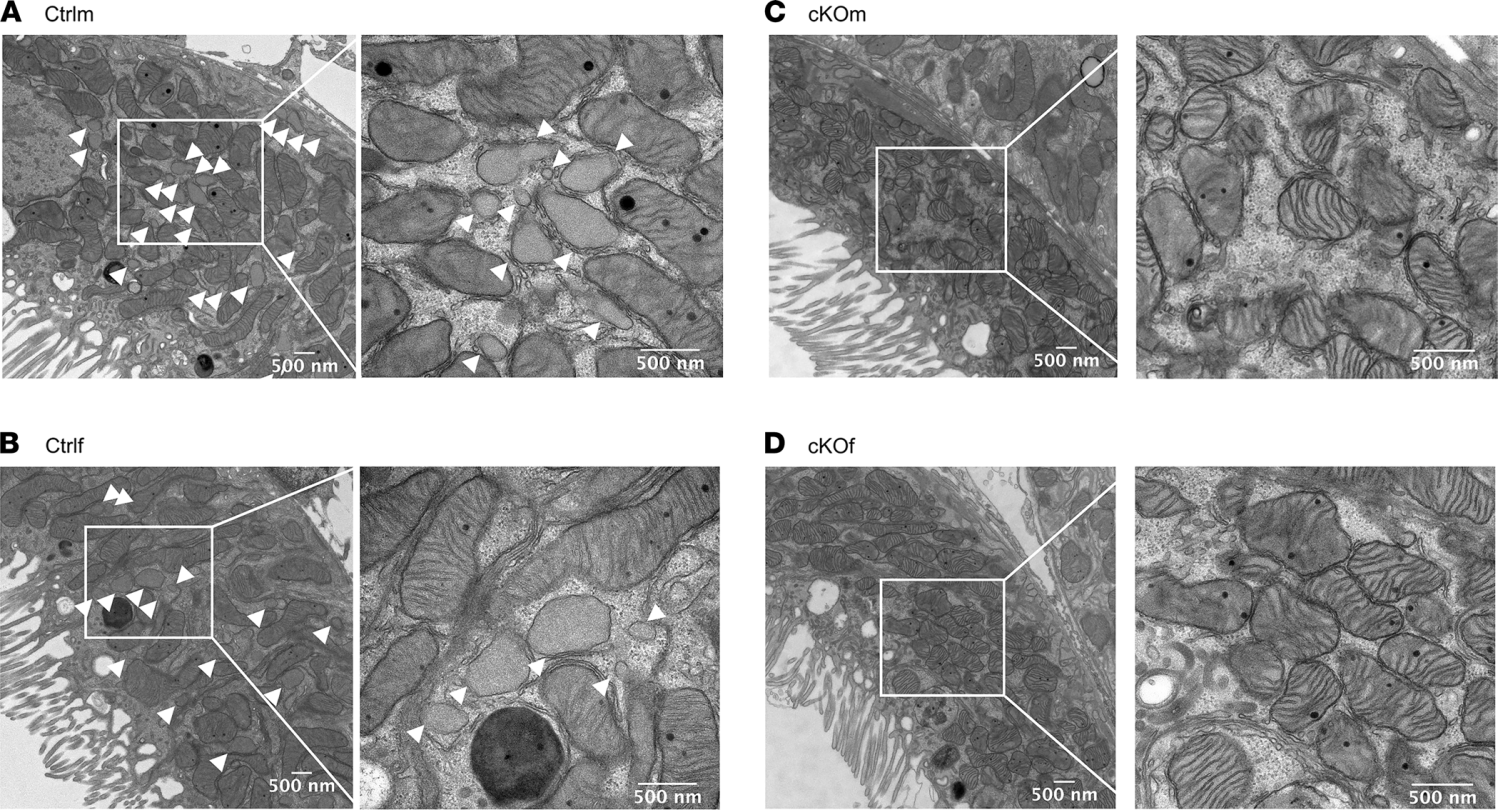
Validation of the cKO model by electron microscopy. (**A**–**D**) Electron microscopy images of kidney proximal tubules in kidney cortex of Ctrlm (**A**), Ctrlf (**B**), cKOm (**C**), and cKOf (**D**) mice. The images are representative of 4 mice/genotype with 15 images analyzed/mice. White arrowheads indicate peroxisomes.

**Figure 3:**
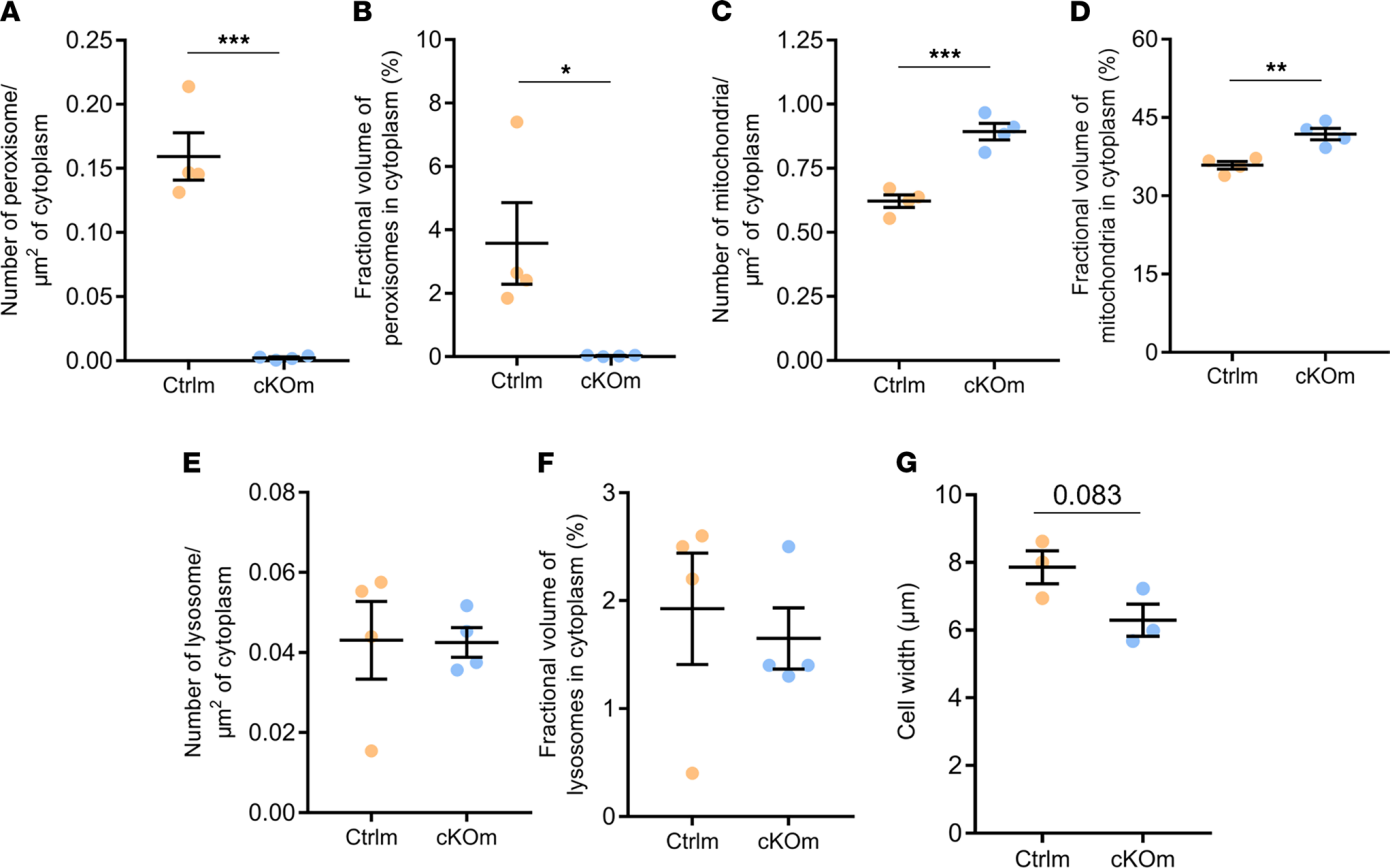
Stereological analysis of proximal tubule cells in kidneys of Ctrlm and cKOm mice. (**A**) Number of peroxisomes/μm^2^ of cytoplasm; (**B**) fractional volume of peroxisomes in percentage of cytoplasm occupied by peroxisomes; (**C**) number of mitochondria/μm^2^ of cytoplasm; (**D**) fractional volume of mitochondria in percentage of cytoplasm occupied by mitochondria; (**E**) number of lysosomes/μm^2^ of cytoplasm; (**F**) fractional volume of lysosomes in percentage of cytoplasm occupied by lysosomes; (**G**) cell width. *P* = 0.083 (**G**). Stereology analysis was performed on *n* = 3–4 mice with 3 kidney cortex pieces per mouse, 15 micrographs per sample. Box and whiskers represent mean ± SEM; unpaired *t* test, ***P < 0.0001, **P < 0.001, *P < 0.05.

Electron microscopy assessment of kidney cortex revealed a high number of peroxisomes in proximal tubule cells of Ctrlm and Ctrlf mice (Figure 2, A and B, respectively). Peroxisomes were virtually absent in proximal tubules of cKOm and cKOf mice (Figure 2, C and D, respectively). Stereological tools were employed for quantitative analysis of cellular organelles and cellular dimensions in proximal tubules of Ctrlm and cKOm mice. Both peroxisomes’ volume density (number/μm^2^ cytoplasm) and the percentage of cytoplasm occupied by peroxisomes in proximal tubule cells were dramatically decreased in cKOm mice (Figure 3, A and B, respectively). Conversely, mitochondria volume density as well as the percentage of cytoplasm occupied by mitochondria in proximal tubule cells were increased in cKOm mice as compared with Ctrlm mice (Figure 3, C and D, respectively). Lysosomes’ volume density and percentage of cytoplasm occupied by lysosomes in proximal tubule cell were not different between cKOm and Ctrlm mice (Figure 3, E and F, respectively). As shown in Figure 3G, proximal tubule cells from cKOm mice had a tendency for reduced cell width (*P* = 0.083).

### Integrated transcriptomic and metabolomic analyses suggest profound reprogramming in metabolic, antioxidant, and lipid synthesis pathways in kidneys of cKO mice.

**Figure 4:**
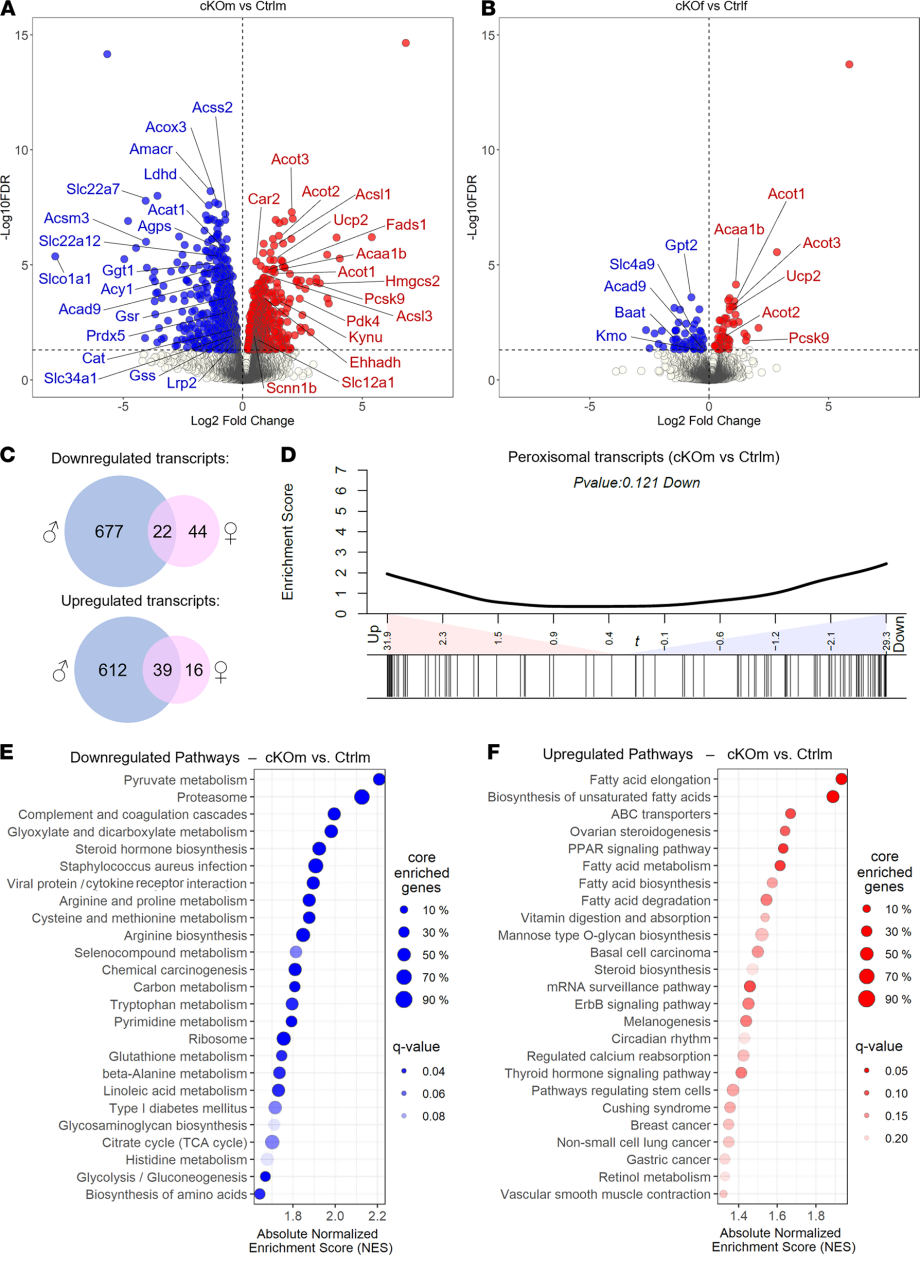
Transcriptional reprogramming in the kidneys of cKO mice. (**A** and **B**) Volcano plot representing the relative transcriptional expression of all renal transcripts in cKOm versus Ctrlm (**A**) or cKOf versus Ctrlf (**B**). Transcripts depicted in blue are significantly downregulated while transcripts depicted in red are significantly upregulated. (**C**) Venn diagrams showing the number of transcripts significantly downregulated or upregulated in cKOm (in blue) or cKOf (in pink) mice versus Ctrl mice of the same sex. A significant transcript regulation is considered when the adjusted *P* value referred to as “FDR” is <0.05. (**D**) Enrichment analysis of a homemade gene set (based on the KEGG pathway mmu04146) targeting 100 transcripts related to peroxisomal functions, in cKOm versus Ctrlm mice. (**E** and **F**) Scatter plot of the top 25 most downregulated (**E**) or upregulated (**F**) metabolic pathways in cKOm versus Ctrlm mice, based on an untargeted GSEA using a database of 543 KEGG metabolic pathways. Pathways are sorted by their absolute normalized enrichment score. A significant pathway regulation can be considered when adjusted *P* value referred to as “q value” is <0.2.

Integrated deep transcriptome-sequencing and metabolome analysis was performed to identify molecular alterations in kidneys of cKO mice (GSE179202). Comparisons of transcriptomes revealed 1350 transcripts differentially expressed in kidneys of Ctrlm and cKOm mice (Figure 4A and Supplemental Table 2, FDR < 5%) and 121 transcripts differentially expressed in kidneys of Ctrlf and cKOf mice (Figure 4B and Supplemental Table 3, FDR < 5%). Sixty-one differentially expressed transcripts were present in both sexes (Figure 4C and Supplemental Figure 3). Enrichment analysis of 100 genes encoding proteins related to peroxisomal function (Kyoto Encyclopedia of Genes and Genomes [KEGG] pathway hsa04146) performed on transcriptomes of Ctrlm and cKOm mice revealed 52 transcripts either up- or downregulated in kidneys of cKOm mice (Figure 4D and Supplemental Figure 4). Gene set enrichment analysis (GSEA) performed on KEGG Pathway database (release of December 11, 2020) showed downregulation of pathways related to metabolism of pyruvate, glyoxylate and dicarboxylate, amino acids (arginine, proline, cysteine, methionine, tryptophan, alanine, and histidine), or glutathione (Figure 4E and Supplemental Figure 5) and upregulation of pathways related to fatty acid synthesis and degradation, PPAR signaling, and ABC transporters (Figure 4F and Supplemental Figure 6). No enrichment was found in pathways linked to inflammation or fibrosis. Targeted analysis of several groups of functionally related genes that are not represented in KEGG pathways revealed substantial changes in expression of genes encoding enzymes involved in peroxisomal metabolism of fatty acids (ACSL1/3/4/6, ACSVL1, ACOT3/4, ACNAT1, ACOX3, ABCD3, EHHADH, and ACAA1B), plasmalogen biosynthesis (FAR1 and AGPS), bile acid synthesis (AMACR and HSD17B4), and reactive oxygen species (ROS) detoxification (CAT and SOD1) (Supplemental Figure 3). Alterations were also noted in expression of a large number of genes critical for membrane transport processes in the proximal tubule, thick ascending limb (TAL), and distal nephron (Supplemental Figure 7 and Supplemental Table 4). For instance, in the proximal tubule, we found a reduction in expression of aquaporin-1 water channel (Aqp1); megalin (Lrp2); phosphate transporter NaPi-2a (Slc34a1); urate transporters Urat1 (Slc22a12), Npt1/4 (Slc17a1/3), and Oat1 (Slc22a6); and numerous other organic anion and amino acid transporters. In the TAL and distal nephron, there was a substantial increase in expression of genes encoding proteins involved in sodium reabsorption: NKCC2 (SLC12A1), CLC-Kb (CLCNKB), βENAC (SCNN1B) (FDR < 0.05), and NCC (SLC12A3) (FDR = 0.05).

**Figure 5:**
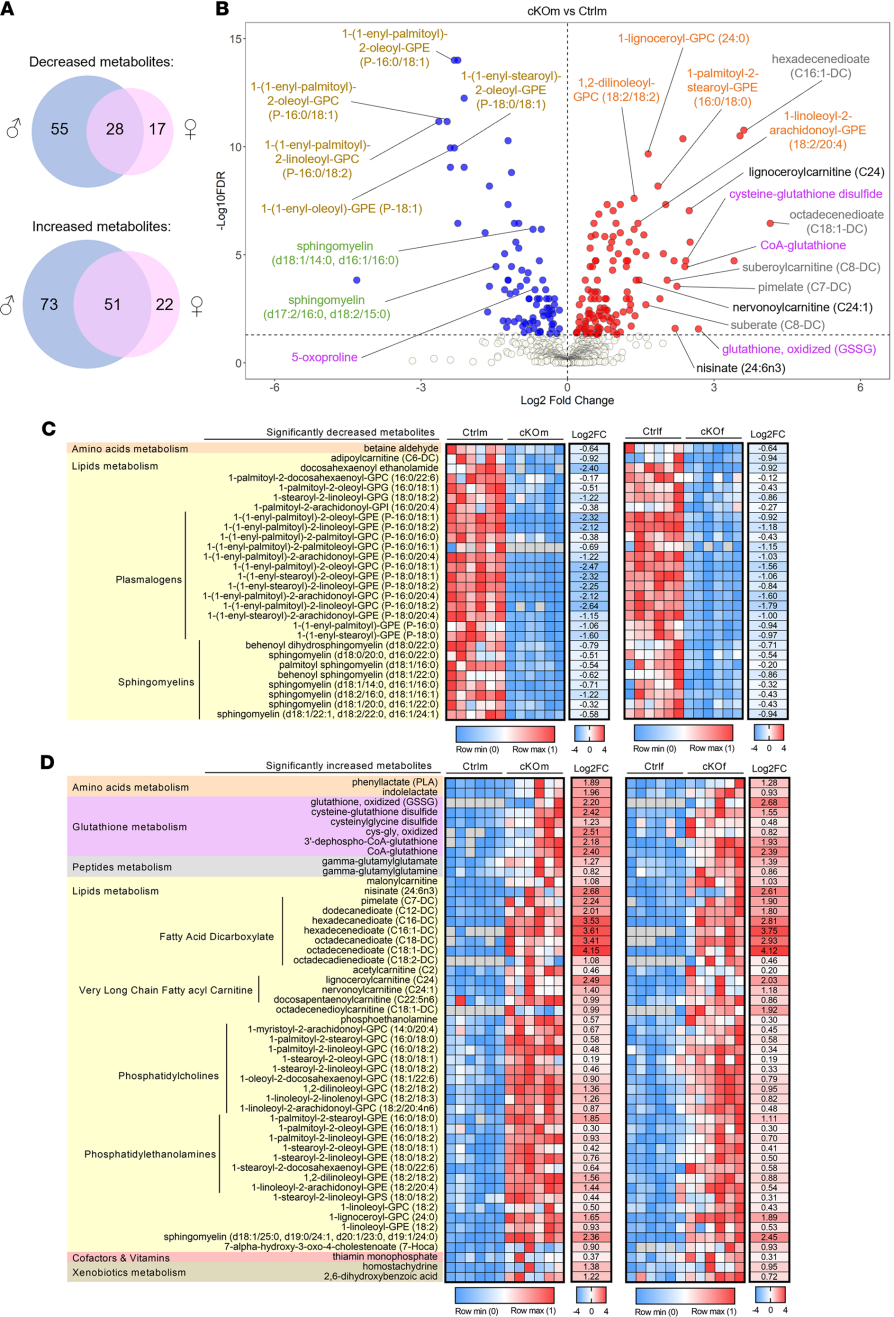
Remodeling of the renal metabolome in cKO mice. (**A**) Venn diagrams representing the number of detected renal metabolites showing a significantly decreased or increased abundance in cKOm (in blue) or cKOf (in pink) mice versus Ctrl mice of the same sex. (**B**) Volcano plot representing the relative abundance of all detected metabolites in kidneys of cKOm versus Ctrlm. Metabolites depicted with blue dots are significantly less abundant, and transcripts depicted with red dots are significantly more abundant in kidneys of cKOm mice as compared with Ctrlm mice. The names of some representative metabolites are depicted using colors shared for related metabolites. (**C** and **D**) Heatmaps of metabolites showing a significantly decreased (**C**) or increased (**D**) abundance in cKO mice of both sexes as compared with Ctrl mice. A significant difference of abundance is considered when adjusted *P* value from a 2-way ANOVA referred to as “FDR” is <0.05. Metabolites are identified by their biochemical name and sorted by related metabolisms and subclasses of metabolites. For each metabolite, the individual expression of 6 Ctrl and 6 cKO mice normalized between 0 and 1 and the log_2_-transformed mean fold change of expression (Log2FC) in cKO versus Ctrl mice are given, for both sexes. For calculation of the mean FC of expression, missing values (depicted in gray) have been replaced by the minimum value of both genotypes from the same sex.

From the total of 852 detected metabolites, 207 showed differential abundance in kidneys of Ctrlm and cKOm mice, and 118 showed differential abundance in kidneys of Ctrlf and cKOf mice (Supplemental Table 5, FDR < 5%). Seventy-nine metabolites demonstrating differential abundance were common in both comparisons (Figure 5A and Supplemental Table 6). Among metabolites showing the most significant difference were plasmalogens and sphingomyelins (decreased abundance) and glutathione-related metabolites and dicarboxylic acids (increased abundance) in kidneys of both cKOm and cKOf mice (Figure 5B and Supplemental Figure 8, respectively). Global analysis of metabolome confirmed that plasmalogens and sphingomyelins constituted a majority of metabolites showing decreased abundance in kidneys of both cKOm and cKOf mice (Figure 5C). LCFA dicarboxylates, very-long-chain fatty acyl-carnitines, phosphatidylcholines, and phosphatidylethanolamines were present among metabolites with increased abundance, in addition to glutathione-related metabolites and dicarboxylic acids (Figure 5D).

**Figure 6:**
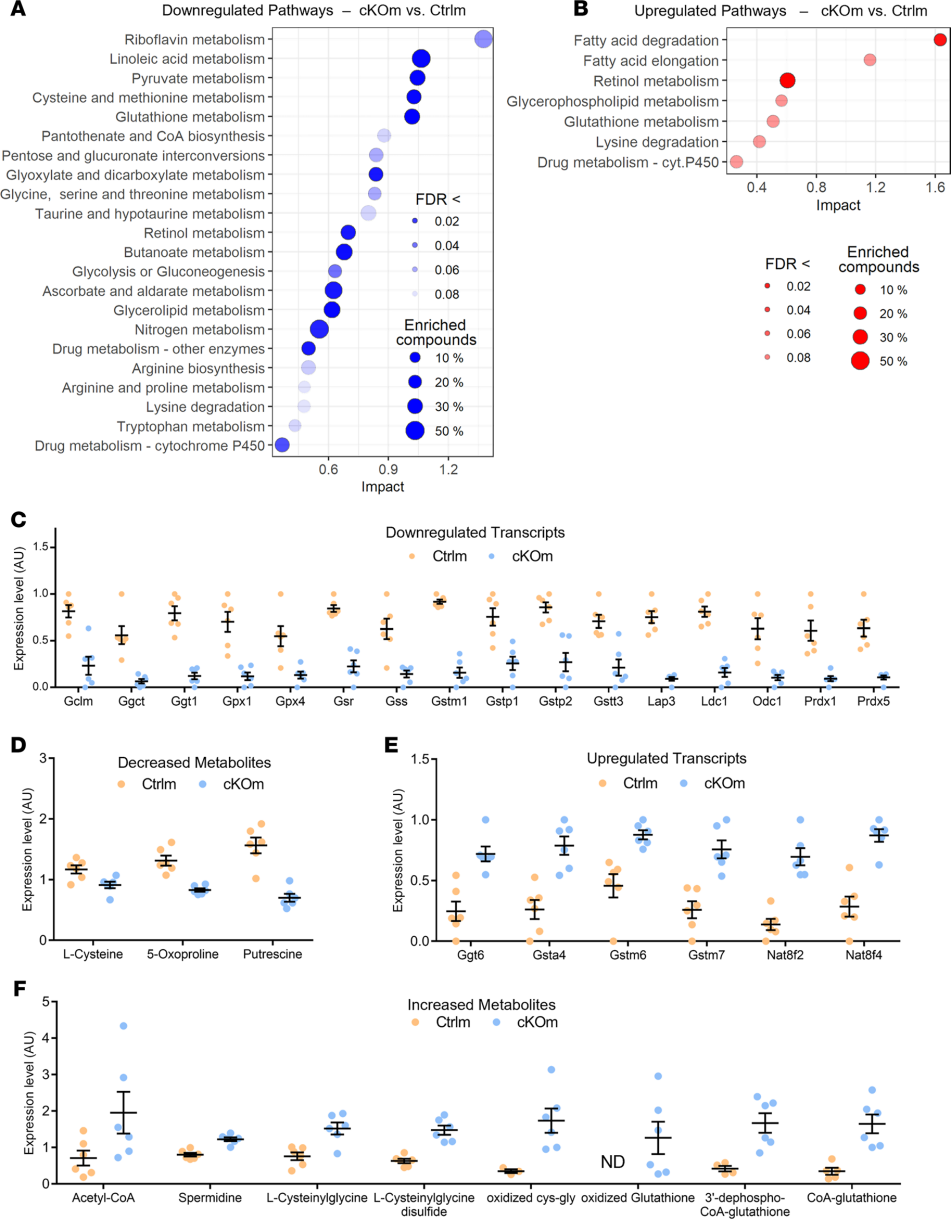
Joint analysis of transcriptional and metabolic changes in cKOm mice. (**A** and **B**) Scatter plot of significantly downregulated (**A**) or upregulated (**B**) metabolic pathways, based on a joint pathway analysis of both regulated transcripts and modulated metabolites in kidneys of cKOm versus Ctrlm mice. Pathways are sorted by their absolute impact, and a significant pathway regulation is considered when adjusted *P* value referred to as “FDR” is <0.1. The size of each dot depends on the percentage of all transcripts and metabolites (“compounds”) of the pathway that are significantly affected in cKOm mice. (**C** and **D**) Relative expression of glutathione-related transcripts (**C**) and metabolites (**D**) significantly less abundant (FDR < 0.05) in cKOm mice as compared with Ctrlm mice. (**E** and **F**) Relative expression of glutathione-related transcripts (**E**) and metabolites (**F**) significantly more abundant (FDR < 0.05) in cKOm mice as compared with Ctrlm mice. Individual values from 6 cKOm and 6 Ctrlm mice are depicted after transformation from raw individual data: values of metabolite abundance have been divided by the median value of both genotypes, while values of transcript expression from both genotypes have been normalized between 0 and 1. Box and whiskers represent the mean and the SEM, respectively. ND, not detected.

Joint pathway analysis (MetaboAnalyst 5.0) of transcripts and metabolites exhibiting increased or decreased abundance in kidneys of cKOm mice compared with Ctrlm mice identified 22 downregulated and 7 upregulated pathways (Figure 6, A and B, respectively; FDR < 0.1; Supplemental Table 7). Nitrogen metabolism, pyruvate metabolism, glycolysis, and glyconeogenesis were identified among the downregulated pathways while fatty acid degradation was identified among the upregulated pathways (Figure 6, A and B, respectively). Glutathione metabolism and retinol metabolism were present among both up- and downregulated pathways (Figure 6, A and B). Detailed analysis of transcripts and metabolites related to glutathione metabolism identified 16 transcripts and 3 metabolites with decreased abundance in kidneys of cKOm mice (Figure 6, C and D, respectively), along with 6 transcripts and 8 metabolites exhibiting increased abundance (Figure 6, E and F, respectively). The oxidized form of glutathione (GSSG) was present in cKOm but not in Ctrlm mice, thereby suggesting oxidative stress in cKOm mice (Figure 6F).

### High-fat feeding challenge does not lead to additional oxidative stress in cKO mice.

**Figure 7:**
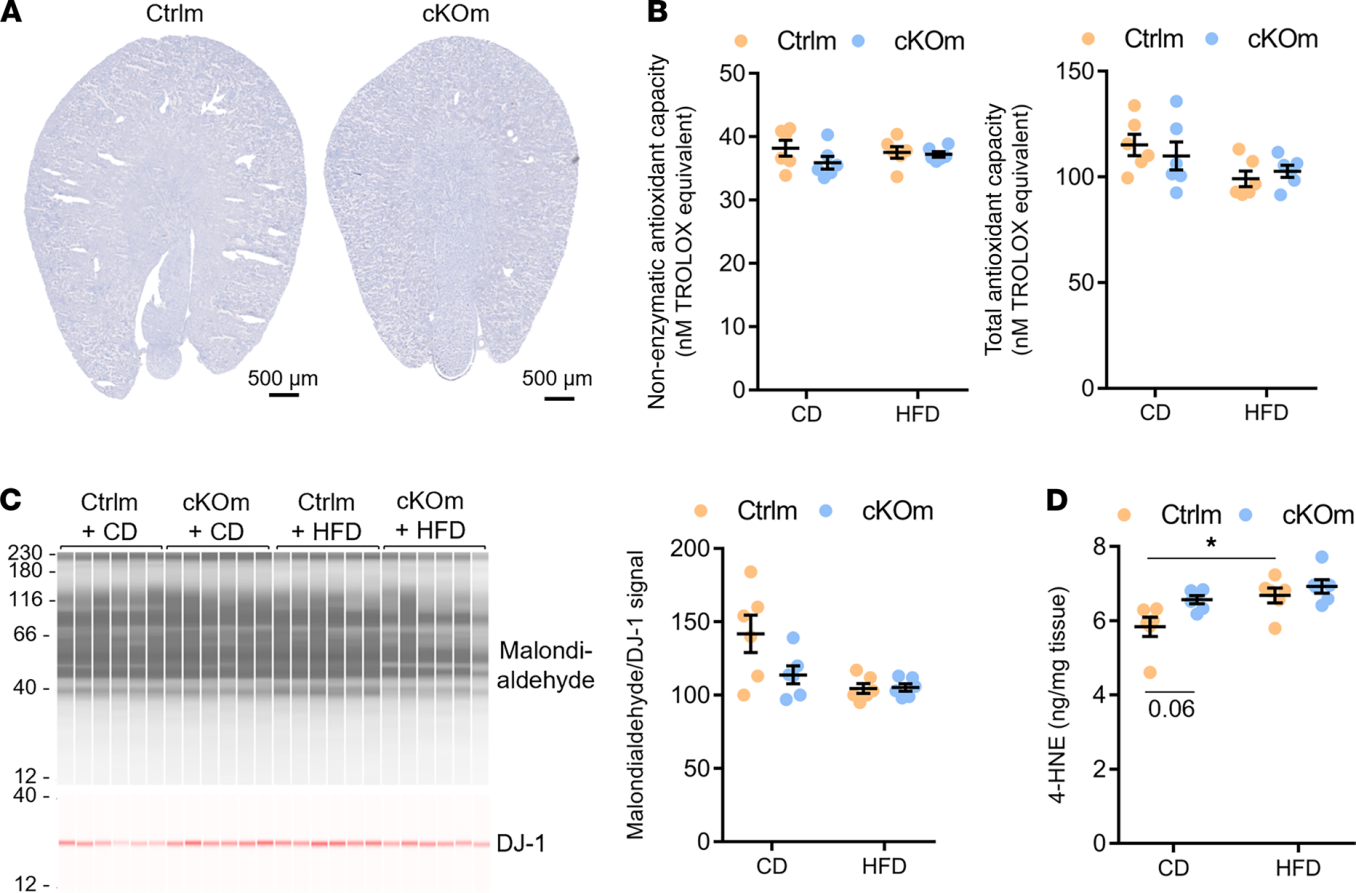
Analysis of the antioxidant capacity and level of lipid peroxidation in cKOm mice fed under HFD. (**A**) Gross morphological structure and Oil Red O staining of neutral lipid depositions (in red) in the kidney of cKOm and Ctrlm adult mice fed under HFD for 4 weeks. The absence of the red color indicates the absence of lipid deposition. (**B**) Results of a Trolox assay performed on renal extracts showing nonenzymatic antioxidant capacity (left panel) and total antioxidant capacity (right panel) of Ctrlm and cKOm mice fed under control diet (CD) or HFD for 4 weeks. (**C**) Immunoblot performed on renal extracts targeting the product of lipid peroxidation malondialdehyde (left panel) and its quantification (right panel) in Ctrlm and cKOm mice fed under CD or HFD for 4 weeks. DJ-1 immunoblot is used as a loading control to normalize malondialdehyde abundance. (**D**) Amount of 4-HNE measured by competitive ELISA in kidney extracts from Ctrlm and cKOm mice fed under CD or HFD for 4 weeks. Box and whiskers represent mean ± SEM. Two-way ANOVA and post hoc Tukey’s multiple comparisons test, *P < 0.05.

Alterations in glutathione-related pathways, depletion of plasmalogens, and decreased expression of catalase suggested oxidative stress and/or rearrangement of different antioxidant defense systems in kidneys of cKOm mice. To test these hypotheses, we challenged cKOm mice with high-fat diet (HFD) for 4 weeks. This challenge did not result in albuminuria but increased urinary volume and urinary excretion of calcium and urate. No difference was observed in tested plasma parameters, including calcemia and uricemia (Supplemental Table 8). No gross morphological changes or lipid accumulation was detected in kidneys of HFD-challenged Ctrlm and cKOm mice (Figure 7A). Total and nonenzymatic antioxidant capacity as assessed by Trolox assay (Figure 7B) as well as tissue levels of malondialdehyde, a marker of polyunsaturated fatty acid peroxidation (Figure 7C), were not different between treatments and genotypes. The abundance of lipid peroxidation product 4-hydroxynonenal (4-HNE) was increased in Ctrlm mice treated with HFD as compared with Ctrlm mice on control diet, but no difference was observed in cKOm mice treated with the 2 diets (Figure 7D).

## Discussion

A high abundance of peroxisomes in the proximal tubule together with the presence of renal abnormalities in patients with ZSD have strongly suggested an important role of peroxisomes in the kidney. Moreover, kidney-specific expression of several peroxisomal enzymes has supported the idea that renal peroxisomes may have biological functions partially distinct from those in other tissues (15). To test these hypotheses, we examined renal function in mice devoid of peroxisomes specifically in the renal tubule. Among different mouse models employed to study the role of peroxisomes, we selected mice allowing conditional genetic inactivation of *Pex5* encoding peroxisome biogenesis factor PEX5 essential for peroxisome formation. This model was chosen because (i) *Pex5*-null mice exhibit multiple biochemical and functional abnormalities reminiscent of those observed in patients with ZSD (11) and (ii) a large spectrum of mouse models with tissue-specific ablation of *Pex5* has been analyzed (16), thereby allowing us to compare the functional consequences of peroxisomal deficiency in the renal tubule with those observed in other tissues. As demonstrated by Baes et al., *Pex5*-null mice exhibited intrauterine growth retardation, malformation of the brain, and severe hypotonia and died within the first 72 hours of life (11). Biochemical analyses revealed several hallmarks of ZSD, including depletion of plasmalogens in liver and brain and strong increase in plasma levels of VLCFA. No cortical renal cysts were found in the kidney of *Pex5*-null mice at P0.5, but retardation in the intrauterine maturation of glomeruli was observed. The presence of calcium oxalate deposits was not investigated.

In cKOm mice, ablation of peroxisomal biogenesis in the renal tubule did not cause any major phenotype, except decreased KW and BW in infant cKOm mice and decreased KW/BW ratio in adult cKOm mice. In cKOf mice, incomplete excision of floxed *Pex5* allele together with much milder effect of *Pex5* deletion on renal transcriptome and metabolome suggested that some residual peroxisomes may remain in the renal tubule. However, sex specificity of peroxisomal functions in the kidney cannot be ruled out as well. We did not find any significant morphological difference in kidneys of cKOm mice that could explain the lower KW or KW/BW ratio. However, the tendency for reduced proximal cell width might be a possible cause of this difference. Importantly, renal homeostatic function was maintained in cKOm mice despite marked changes in expression levels of transcripts encoding proteins involved in transepithelial transport of ions, water, amino acids, organic anions and cations, phosphate, urate and in the megalin-mediated multiligand endocytic pathway. Of note, a majority of downregulated transcripts encoded transporters expressed in the proximal tubule whereas most of the upregulated transcripts encoded transporters expressed in more distal parts of the nephron. For instance, increased expression of Nkcc2, Clc-Kb, Ncc, and βENaC transcripts suggested a compensatory upregulation in postproximal sodium reabsorption. The integrated analysis of renal transcriptome and metabolome revealed profound changes in a variety of cellular pathways related to cellular metabolism, lipid membrane composition, and redox homeostasis. In kidneys of cKOm mice, about 8% of transcriptome and about 24% of detected metabolites exhibited different levels as compared with Ctrlm mice. As expected, many of these transcripts and metabolites were related to peroxisomal function. A dramatic reduction was observed in abundance of ether phospholipid plasmalogens that are synthesized in peroxisomes (~67% in cKOm mice and ~50% in cKOf mice, unweighted sum of all detected plasmalogen species). Plasmalogens are major cell membrane phospholipids with a growing number of attributed functions, including membrane integrity, cellular signaling, and antioxidant defense. In the kidney, plasmalogens represent approximately 20% of total phospholipids (17), thus suggesting that at least 10% of the phospholipid composition differed in kidneys of cKOm and cKOf mice from respective controls. The depletion of plasmalogens was potentially compensated by increased abundance of structurally similar phosphatidylethanolamines (PEs) and by phosphatidylcholines (PCs), which exhibited a substantial enrichment in kidneys of both cKOm and cKOf mice. Of note, a similar compensatory increase of PEs and PCs was observed in kidneys of patients with ZSD (17).

The metabolism of the proximal tubule mainly relies on fatty acid oxidation, which is characterized by high generation of ROS produced both in mitochondria and peroxisomes. In cKO mice, pathways related to fatty acids’ degradation/biosynthesis were substantially upregulated, potentially suggesting compensatory enhancement of mitochondrial energy production from these substrates. Since one of the main functions of peroxisomes is detoxification of ROS, we hypothesized that ablation of peroxisome biogenesis would induce oxidative stress in kidneys of cKO mice. However, molecular analyses did not reveal changes in the antioxidant capacity and biomarkers of oxidative stress. Challenge with HFD did not induce alterations in functional phenotype, except mild increase in urinary volume and urinary excretion of calcium and urate in cKOm mice. Integrated transcriptome and metabolome analysis allowed identification of the glutathione pathway as a possible compensatory mechanism for the maintenance of the overall antioxidant capacity in renal proximal tubule cells lacking peroxisomes.

Conditional deletion of *Pex5* in the fetal liver (12), in pancreatic β cells (18), and in different cell types of the central nervous system (reviewed in ref. 16) causes variable but mostly serious organ-specific functional deteriorations. This was not the case in the kidney. In this study we identified several intrinsic renal mechanisms that potentially allow coping with the lack of peroxisomes. As the kidney is the organ that exhibits high blood perfusion rate, another possibility lies in the extrarenal origin of at least a part of metabolites that the kidney does not produce in the absence of peroxisomes but that are required for normal renal function. Collectively, our data suggest that renal tubular peroxisomes are dispensable for normal renal function and that pathophysiological changes in kidneys of patients with ZSD are of extrarenal origin.

## Methods

### Animals.

The procedures used to generate the *Pex5*^lox/lox^/*Pax8-rtTA*/LC-1 Cre mice were described previously (13). The 3 mouse lines used in this study are inbred strains, bred on the genetic background of the C57BL/6J mouse (The Jackson Laboratory). Before all experiments, mice were adapted to a 12-hour light/12-hour dark cycle. All experiments were performed at circadian time ZT3 (ZT0 is the time when the light is switched on, and ZT12 is the time when the light is switched off).

*Pex5*^lox/lox^/*Pax8-rtTA*/LC1 triple transgenic male or female mice (referred to as cKOm and cKOf) and *Pex5*^lox/lox^ mice (referred to as Ctrlm or Ctrlf) were used.

Pex5 recombination in newborns was performed by addition of 0.2% DOX and 2% sucrose in the drinking water of pregnant female mice for 2 weeks, starting from their 14th gestation day. For Pex5 recombination in adult mice, DOX and sucrose were added in drinking water of 8-week-old mice. The renal structure and function were evaluated 4 weeks after the end of the DOX treatment in 4-week-old infant mice or 14-week-old adult mice. After their weaning at 3 weeks, mice were fed with a standard control diet containing 4.2% of fat (Ssniff low-fat control diet) or when mentioned, for 4 weeks with a matching HFD containing 20.7% of fat (mostly LCFAs).

### Metabolic cages and blood and urine chemistry.

Mice were housed in individual metabolic cages (Tecniplast). Urine collection was performed after a 3-day adaptation period. Urine and blood chemistry was analyzed as previously described (19). Plasma and urine composition was measured in the Laboratoire Central de Chimie Clinique, Centre Hospitalier Universitaire Vaudoise University Hospital (Lausanne, Switzerland).

### Electron microscopy.

Kidney samples were cut in pieces around 1 mm^3^ and fixed in glutaraldehyde solution (EMS) 2.5% in phosphate buffer (PB, 0.1 M pH 7.4) (MilliporeSigma) during 2 hours at room temperature (RT). Then, they were rinsed 3 times, 5 minutes each, in PB buffer and then postfixed by a fresh mixture of osmium tetroxide 1% (EMS) with 1.5% of potassium ferrocyanide (MilliporeSigma) in PB for 2 hours at RT. The samples were then washed 3 times in distilled water and dehydrated in acetone solution (MilliporeSigma) at graded concentrations (30% 90 minutes; 70% 90 minutes; 100% 120 minutes; 100% 240 minutes). This was followed by infiltration in Epon resin (MilliporeSigma) at graded concentrations (Epon/acetone [1/3; v/v] 4 hours; Epon/acetone [3/1; v/v] 4 hours, Epon/acetone [1/1; v/v] 8 hours; Epon/acetone [1/1; v/v] 24 hours) and finally polymerization for 48 hours at 60°C in an oven. Ultrathin sections of 50 nm were cut on a Leica Ultracut (Leica Mikrosysteme GmbH) and picked up on a copper slot grid, 2 × 1 mm (EMS), coated with a polystyrene film (MilliporeSigma). Sections were poststained with uranyl acetate (MilliporeSigma) 2% in H_2_O for 10 minutes, rinsed several times with H_2_O followed by Reynolds lead citrate for 10 minutes, and rinsed several times with H_2_O.

### Stereology.

For stereology analysis, 3 kidney cortex pieces per mouse were analyzed, 15 micrographs per sample with a pixel size of 4.08 nm, using a systematic uniform random sampling with a transmission electron microscope (Philips CM100, Thermo Fisher Scientific) at an acceleration voltage of 80 kV with a TVIPS TemCam-F416 digital camera (TVIPS GmbH).

### Transcriptomic analysis.

All sequencing data are publicly available through NIH Gene Expression Omnibus (accession number GSE179202). RNA-Seq libraries were prepared using 200 ng of total kidney RNA as described in Nikolaeva et al. (19). Illumina TruSeq SR Cluster Kit v4 reagents were used. Sequencing data were processed using Mus musculus.GRCm38.86 gene annotation. Statistical analysis was performed in R (version 3.4.0). Transcripts with low counts were filtered out according to the rule of 1 count per million (cpm) in at least 1 sample. Library sizes were scaled using TMM normalization and log-transformed into cpm using voom (20). Principal component analysis showed a small variability between replicate samples. Differential expression between cKO and Ctrl mice was computed into 1 *F* test using limma (21). *P* values were adjusted as described (22) into FDR values to consider for multiple comparisons, using results of all transcripts in males and females or results of selected transporters or peroxisomal related transcripts in males only. Transcripts with an FDR < 5% were considered significant. Comparisons of expression level were done using mean FC of cpm in cKO mice as compared with Ctrl mice. For graphical representation of individual values in box-and-whisker plots, individual values were normalized between 0 and 1. A weighted GSEA was performed using the unfiltered transcript list ranked by signed *P* value (product of *P* value and sign of the FC). The analyses were performed using the gseKEGG function from the R package clusterProfiler (version 3.16.1) (23). KEGG pathway collections were restricted to gene sets with a minimum and maximum sizes of 10 and 500, respectively. The enrichment scores were normalized by gene set size, and their statistical significance was assessed by permutation tests (*n* = 1000).

### Metabolomic analysis.

Metabolites from frozen kidney samples were identified by ultrahigh-performance liquid chromatography–tandem mass spectroscopy and quantified using AUC (Metabolon Inc). Raw values were log-transformed and normalized in terms of raw area counts. Two-way ANOVA contrast tests were used to identify biochemicals that differed significantly between cKO and Ctrl mice of both sexes. *P* values were adjusted as described (22) into FDR values to consider for multiple comparisons, and metabolites with FDR < 5% were considered significantly modulated. For Log2FC calculation, missing values, if any, were replaced with the minimum observed value in mice of the same sex, for each compound. For graphical representation of individual values in box-and-whisker plots, normalized values were divided by the median value obtained in mice of the same sex, for each compound.

### Joint transcriptome-metabolome analysis.

An integrated metabolic pathway analysis combining results obtained from gene expression and metabolomics studies was conducted using the Joint Pathway Analysis module on the MetaboAnalyst 5.0 website (24). Compound name of metabolites and official gene symbol of transcripts significantly more or less abundant (FDR < 0.05), along with their respective FCs in cKOm mice as compared with Ctrlm mice, were inputs. The integration was performed using KEGG metabolic pathways with default settings (hypergeometric test for overrepresentation analysis, degree centrality for pathway topology analysis, and tight integration by combining queries). Pathways with FDR < 0.1 were considered significantly affected in cKOm mice.

### Stainings.

Stainings of global kidney structure, calcium oxalate deposits, or neutral lipid deposition were performed on transverse kidney slices that were 5 μm thick. Except for neutral lipid staining, mice were anesthetized, and their left kidney was perfused through the abdominal aorta with a 4% paraformaldehyde solution before tissue harvesting. For analysis of global kidney structure, kidney slices embedded in paraffin were deparaffinized, rehydrated, stained with H&E, dehydrated, and mounted in ROTI Histokitt (Carl Roth GmbH). Neutral lipids were stained in kidney slices embedded in OCT compound (Tissue-Tek) using an Oil Red O solution. Calcium oxalate deposits were stained according to the method described by Pizzolato (25). Briefly, kidney slices embedded in paraffin were deparaffinized and rehydrated, then submerged in a 1:1 solution of silver nitrate 5% w/v and hydrogen peroxide 30% and placed under a 60 W light bulb for 30 minutes. Slides were thoroughly washed in distilled water and counterstained with Nuclear Fast Red (MilliporeSigma), then dehydrated before mounting in ROTI Histokitt. Longitudinal kidney slices from a mouse treated with a calcium oxalate nephropathy-inducing diet (1.5% calcium + 1.5% hydroxyproline mixed in standard chow for 3 weeks) served as a positive control of calcium oxalate kidney deposits. Results were analyzed using a Zeiss AxioScan.Z1 Slide Scanner at 100× or 200× original magnification.

### ELISA and Trolox assay.

4-HNE was measured using Lipid Peroxidation (4-HNE) Assay Kit from Abcam (ab238538). Total and nonenzymatic antioxidant capacities were assessed by Trolox assay kit from Abcam (ab65329).

### Statistics.

All data are expressed as mean ± SEM. Statistical tests and threshold for significance are described in figure legends or appropriate Methods sections. Statistical analysis was performed using R packages or GraphPad Prism software version 8.2.1. All *t* tests were 2 tailed.

### Study approval.

All experiments with animals were performed in accordance with the Swiss guidelines for animal care, which conform to the National Institutes of Health animal care guidelines, and approved by Swiss cantonal (Canton de Vaud) and federal veterinary authorities (authorization 31827 to DF) (Direction générale de l’agriculture, de la viticulture et des affaires vétérinaires, Epalinges, Switzerland).

Prior publication: A part of this work has been submitted as an abstract to the 2021 Annual Meeting of the American Society of Nephrology (November 4, 2021. https://www.asn-online.org/education/kidneyweek/2021/program-abstract.aspx?controlId=3605774).

## Author contributions

CA, YB, MB, and DF designed the study; CA, GC, AG, YB, DH, SK, and JD carried out experiments; CA, GC, SP, YB, and DF analyzed the data; CA, YB, and DF made the figures; and DF and YB wrote the manuscript. All authors approved the final version of the manuscript.

## Supplementary Material

Supplemental data

Supplemental table 1

Supplemental table 2

Supplemental table 3

Supplemental table 4

Supplemental table 5

Supplemental table 6

Supplemental table 7

Supplemental table 8

## Version 1 02/22/2022

Electronic publication
