# Supplementary material for: Renal tubular peroxisomes are dispensable for normal kidney function
Source: JCI Insight. 2022 Feb 22;7(4):e155836. doi: 10.1172/jci.insight.155836 (PMC8876468; doi:10.1172/jci.insight.155836)
Supplement: Supplemental data [file jciinsight-7-155836-s069.pdf]

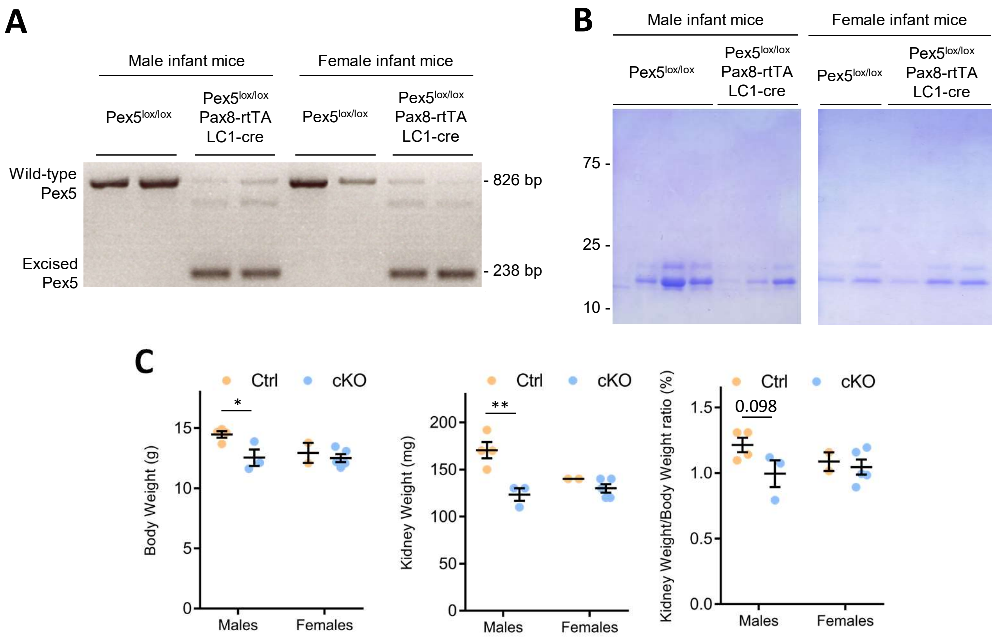
A
Male infant mice
Female infant mice
Pex5lox/lox
Pex5lox/lox Pax8-rtTA LC1-cre
Wild-type Pex5
Excised Pex5
826 bp
238 bp
B
75
25
10
C
Ctrl
cKO
Body Weight (g)
Kidney Weight (mg)
Kidney Weight/Body Weight ratio (%)
0.098
Males
Females

Fig supp. 1

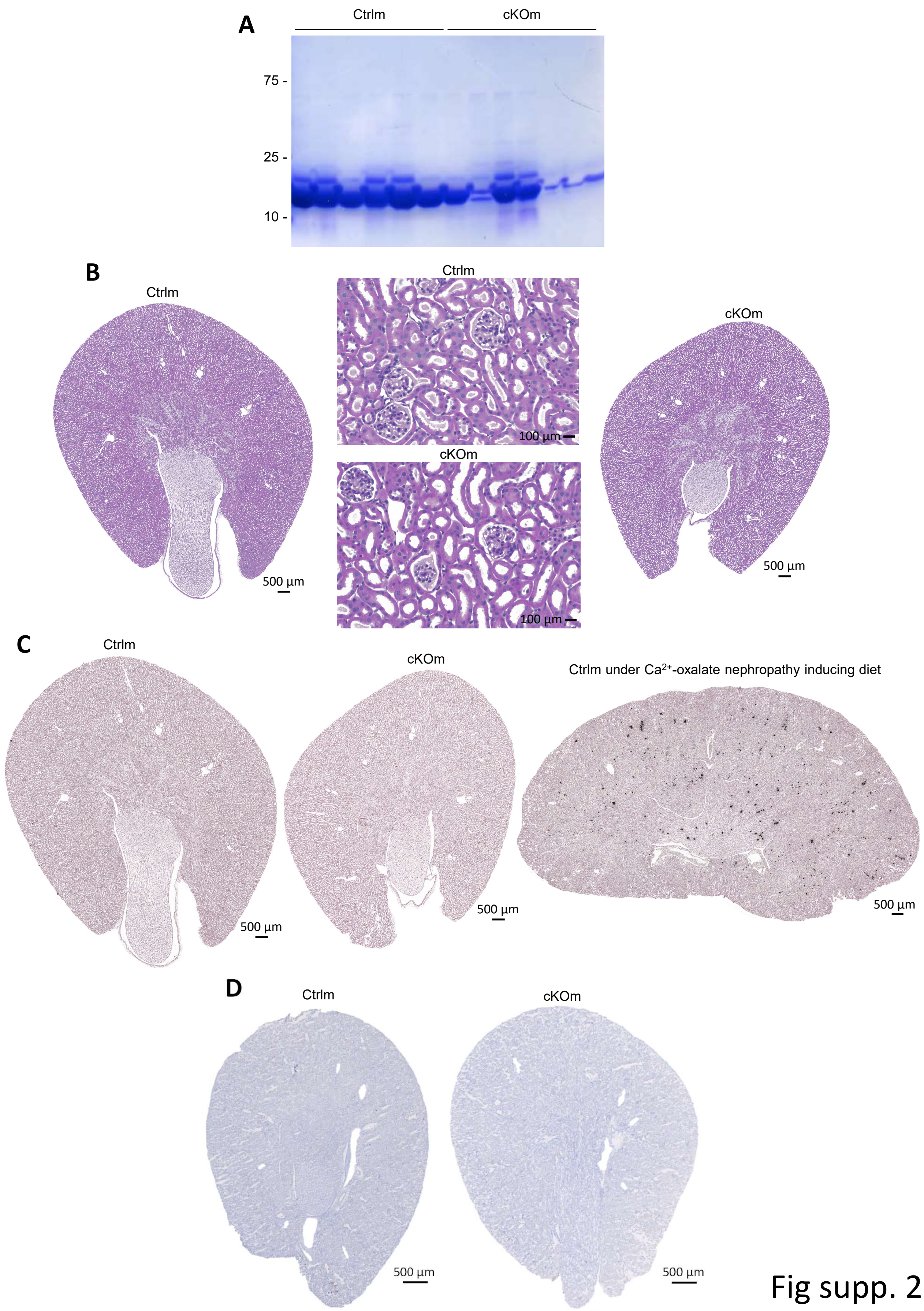
A
Ctrlm
cKOm
75 -
25 -
10 -
B
Ctrlm
Ctrlm
cKOm
cKOm
100 µm
100 µm
500 µm
500 µm
C
Ctrlm
cKOm
Ctrlm under Ca2+-oxalate nephropathy inducing diet
500 µm
500 µm
500 µm
D
Ctrlm
cKOm
500 µm
500 µm

Fig supp. 2

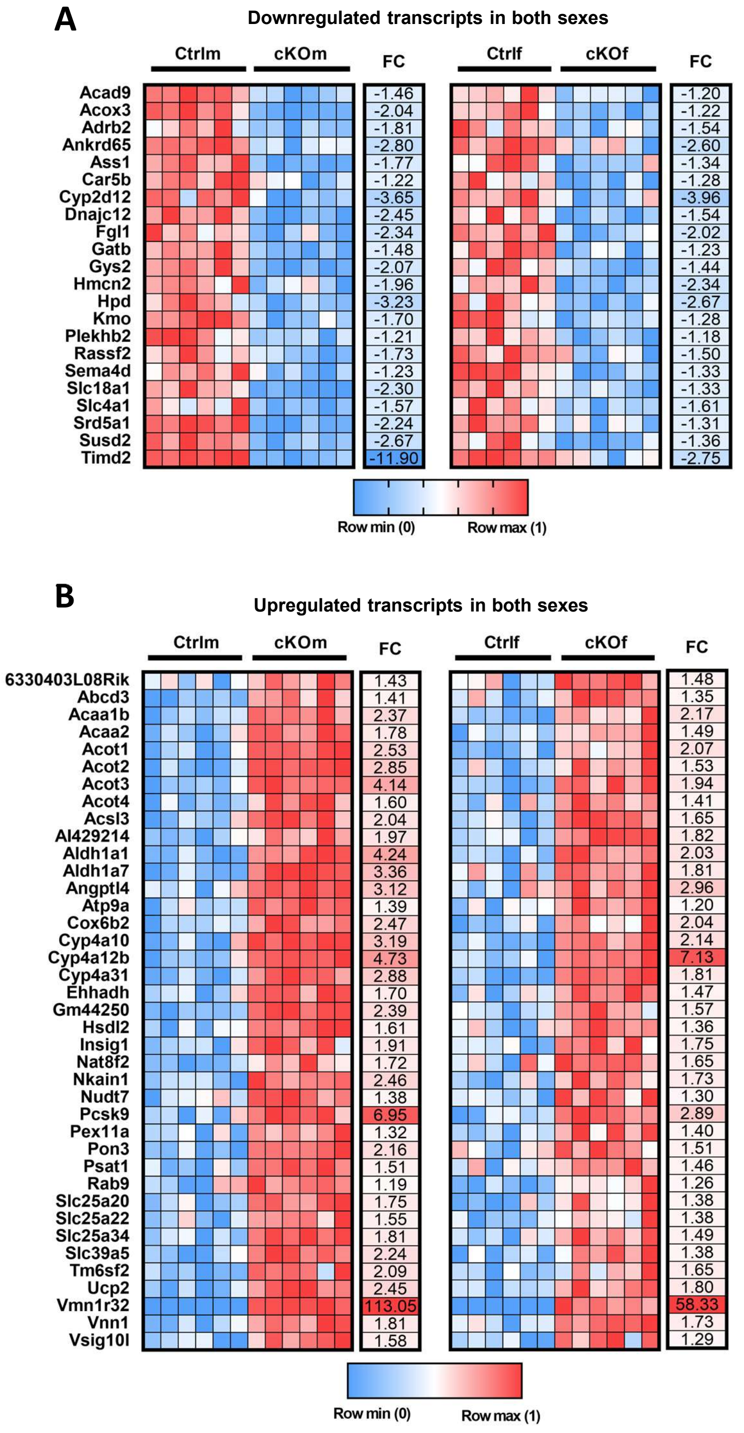
A
Downregulated transcripts in both sexes
Ctrlm
cKOm
FC
Ctrlf
cKOf
FC
Acad9 -1.46 -1.20
Acox3 -2.04 -1.22
Adrb2 -1.81 -1.54
Ankrd65 -2.80 -2.60
Ass1 -1.77 -1.34
Car5b -1.22 -1.28
Cyp2d12 -3.65 -3.96
Dnajc12 -2.45 -1.54
Fgl1 -2.34 -2.02
Gatb -1.48 -1.23
Gys2 -2.07 -1.44
Hmcn2 -1.96 -2.34
Hpd -3.23 -2.67
Kmo -1.70 -1.28
Plekhb2 -1.21 -1.18
Rassf2 -1.73 -1.50
Sema4d -1.23 -1.33
Slc18a1 -2.30 -1.33
Slc4a1 -1.57 -1.61
Srd5a1 -2.24 -1.31
Susd2 -2.67 -1.36
Timd2 -11.90 -2.75
Row min (0)
Row max (1)
B
Upregulated transcripts in both sexes
Ctrlm
cKOm
FC
Ctrlf
cKOf
FC
6330403L08Rik 1.43 1.48
Abcd3 1.41 1.35
Acaa1b 2.37 2.17
Acaa2 1.78 1.49
Acot1 2.53 2.07
Acot2 2.85 1.53
Acot3 4.14 1.94
Acot4 1.60 1.41
Acsl3 2.04 1.65
AI429214 1.97 1.82
Aldh1a1 4.24 2.03
Aldh1a7 3.36 1.81
Angptl4 3.12 2.96
Atp9a 1.39 1.20
Cox6b2 2.47 2.04
Cyp4a10 3.19 2.14
Cyp4a12b 4.73 7.13
Cyp4a31 2.88 1.81
Ehhadh 1.70 1.47
Gm44250 2.39 1.57
Hsdl2 1.61 1.36
Insig1 1.91 1.75
Nat8f2 1.72 1.65
Nkain1 2.46 1.73
Nudt7 1.38 1.30
Pcsk9 6.95 2.89
Pex11a 1.32 1.40
Pon3 2.16 1.51
Psat1 1.51 1.46
Rab9 1.19 1.26
Slc25a20 1.75 1.38
Slc25a22 1.55 1.38
Slc25a34 1.81 1.49
Slc39a5 2.24 1.38
Tm6sf2 2.09 1.65
Ucp2 2.45 1.80
Vmn1r32 113.05 58.33
Vnn1 1.81 1.73
Vsig10l 1.58 1.29
Row min (0)
Row max (1)

Fig supp. 3

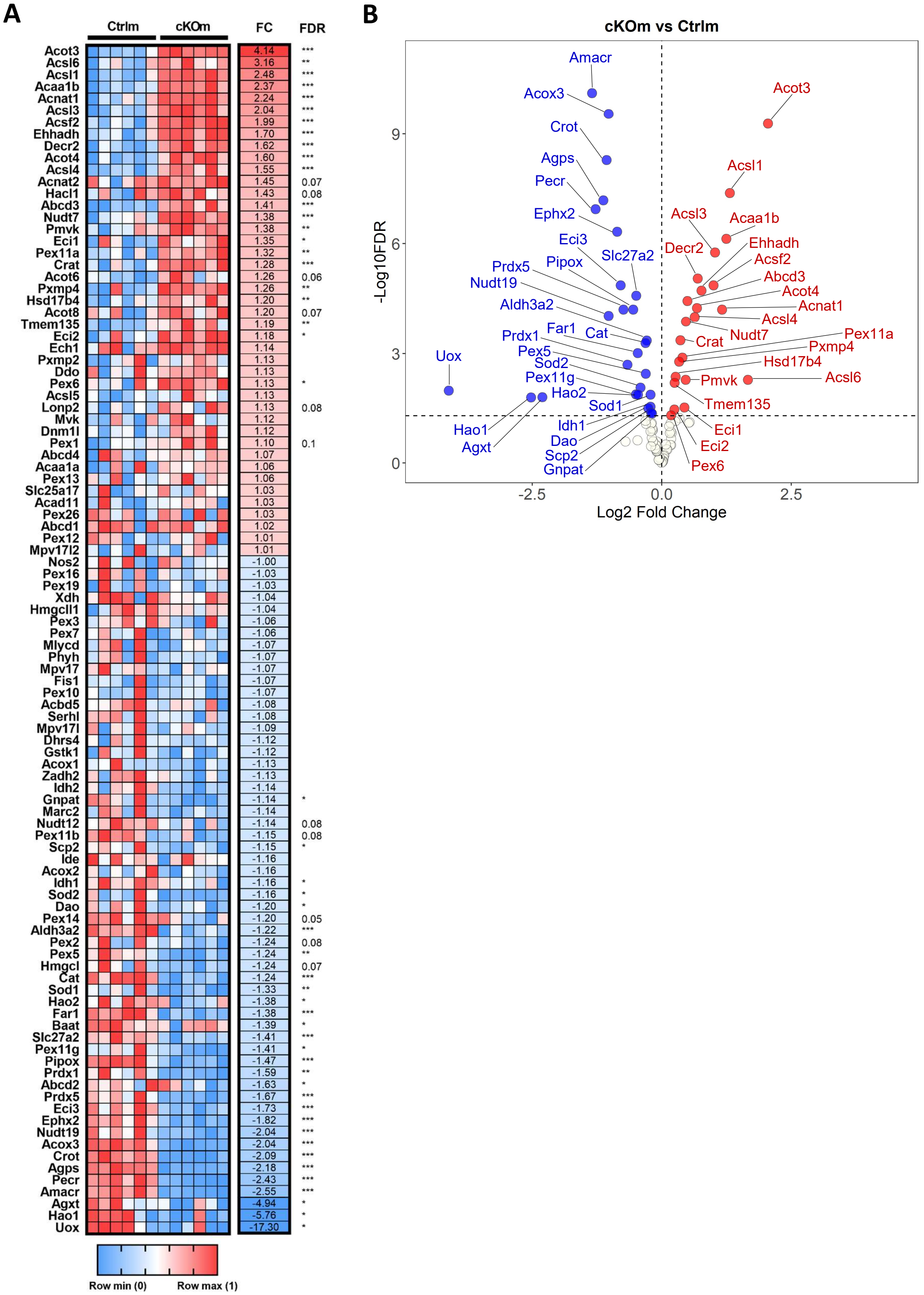
A
Ctrlm
cKOm
FC
FDR
Acot3 4.14 ***
Acsl6 3.16 **
Acsl1 2.48 ***
Acaa1b 2.37 ***
Acnat1 2.24 ***
Acsl3 2.04 ***
Acsf2 1.99 ***
Ehhadh 1.70 ***
Decr2 1.62 ***
Acot4 1.60 ***
Acsl4 1.55 ***
Acnat2 1.45 0.07
Hacl1 1.43 0.08
Abcd3 1.41 ***
Nudt7 1.38 ***
Pmvk 1.38 **
Eci1 1.35 *
Pex11a 1.32 **
Crat 1.28 ***
Acot6 1.26 0.06
Pxmp4 1.26 **
Hsd17b4 1.20 **
Acot8 1.20 0.07
Tmem135 1.19 **
Eci2 1.18 *
Ech1 1.14
Pxmp2 1.13
Ddo 1.13
Pex6 1.13 *
Acsl5 1.13
Lonp2 1.13 0.08
Mvk 1.12
Dnm1l 1.12
Pex1 1.10 0.1
Abcd4 1.07
Acaa1a 1.06
Pex13 1.06
Slc25a17 1.03
Acad11 1.03
Pex26 1.03
Abcd1 1.02
Pex12 1.01
Mpv17l2 1.01
Nos2 -1.00
Pex16 -1.03
Pex19 -1.03
Xdh -1.04
Hmgcll1 -1.04
Pex3 -1.06
Pex7 -1.06
Mlycd -1.07
Phyh -1.07
Mpv17 -1.07
Fis1 -1.07
Pex10 -1.07
Acbd5 -1.08
Serhl -1.08
Mpv17l -1.09
Dhrs4 -1.12
Gstk1 -1.12
Acox1 -1.13
Zadh2 -1.13
Idh2 -1.14
Gnpat -1.14 *
Marc2 -1.14
Nudt12 -1.14 0.08
Pex11b -1.15 0.08
Scp2 -1.15 *
Ide -1.16
Acox2 -1.16
Idh1 -1.16 *
Sod2 -1.16 *
Dao -1.20 *
Pex14 -1.20 0.05
Aldh3a2 -1.22 ***
Pex2 -1.24 0.08
Pex5 -1.24 **
Hmgcl -1.24 0.07
Cat -1.24 ***
Sod1 -1.33 **
Hao2 -1.38 *
Far1 -1.38 ***
Baat -1.39 *
Slc27a2 -1.41 ***
Pex11g -1.41 *
Pipox -1.47 ***
Prdx1 -1.59 **
Abcd2 -1.63 *
Prdx5 -1.67 ***
Eci3 -1.73 ***
Ephx2 -1.82 ***
Nudt19 -2.04 ***
Acox3 -2.04 ***
Crot -2.09 ***
Agps -2.18 ***
Pecr -2.43 ***
Amacr -2.55 ***
Agxt -4.94 *
Hao1 -5.76 *
Uox -17.30 *
Row min (0)
Row max (1)
B
cKOm vs Ctrlm
-Log10FDR
Log2 Fold Change
-2.5
0.0
2.5
0
3
6
9
Amacr
Acox3
Crot
Agps
Pecr
Ephx2
Eci3
Slc27a2
Pipox
Prdx5
Nudt19
Aldh3a2
Far1
Cat
Prdx1
Pex5
Sod2
Uox
Pex11g
Hao2
Sod1
Idh1
Dao
Scp2
Gnpat
Hao1
Agxt
Acot3
Acsl1
Acsl3
Acaa1b
Ehhadh
Decr2
Acsf2
Abcd3
Acot4
Acnat1
Acsl4
Nudt7
Crat
Pex11a
Pxmp4
Hsd17b4
Acsl6
Pmvk
Tmem135
Eci1
Eci2
Pex6

Fig supp. 4

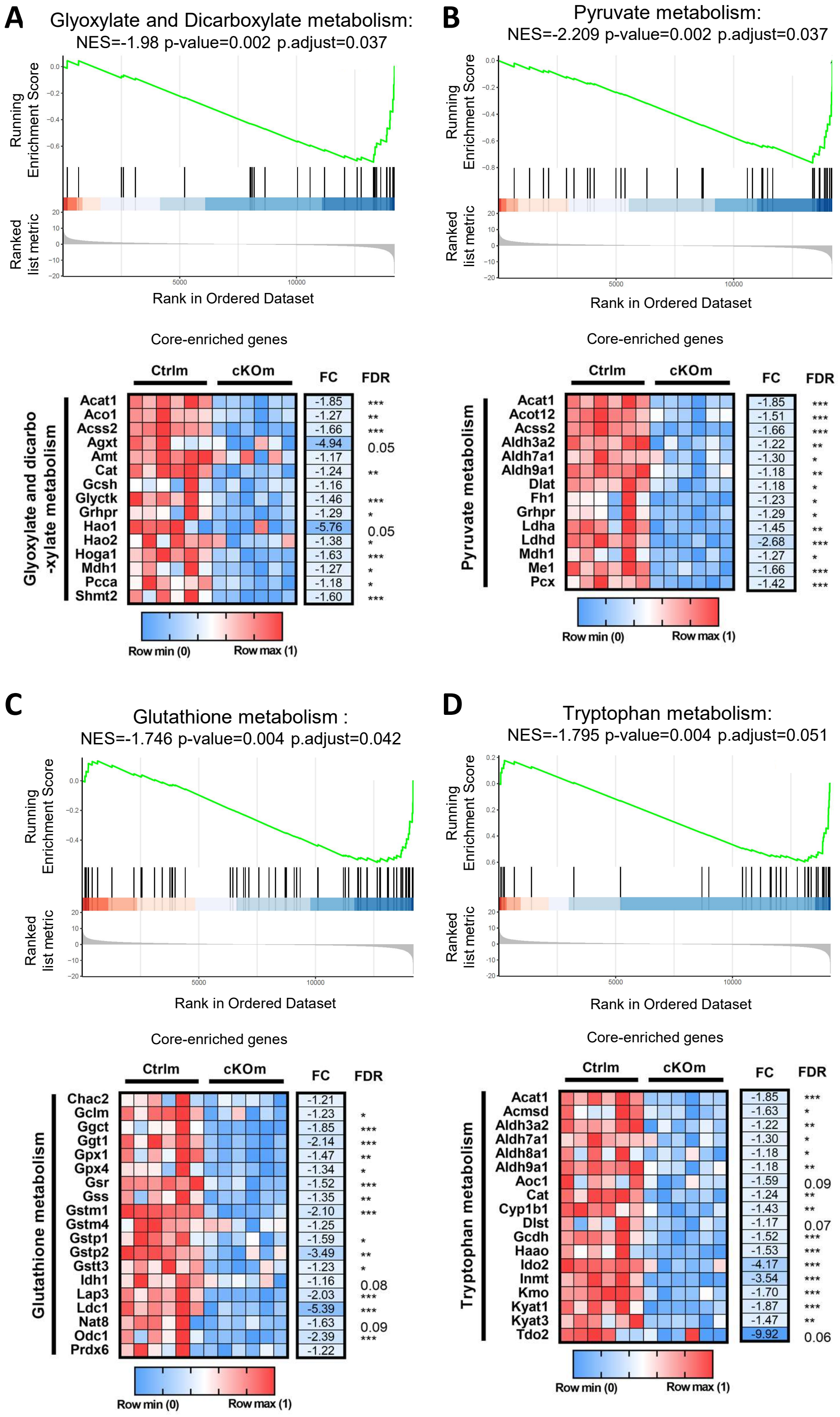
A
Glyoxylate and Dicarboxylate metabolism:
NES=-1.98 p-value=0.002 p.adjust=0.037
Running Enrichment Score
Ranked list metric
Rank in Ordered Dataset
Core-enriched genes
Ctrlm
cKOm
FC
FDR
Glyoxylate and dicarbo -xylate metabolism
Acat1 -1.85 ***
Aco1 -1.27 **
Acss2 -1.66 ***
Agxt -4.94 0.05
Amt -1.17
Cat -1.24 **
Gcsh -1.16
Glyctk -1.46 ***
Grhpr -1.29 *
Hao1 -5.76 0.05
Hao2 -1.38 *
Hoga1 -1.63 ***
Mdh1 -1.27 *
Pcca -1.18 *
Shmt2 -1.60 ***
Row min (0)
Row max (1)
B
Pyruvate metabolism:
NES=-2.209 p-value=0.002 p.adjust=0.037
Running Enrichment Score
Ranked list metric
Rank in Ordered Dataset
Core-enriched genes
Ctrlm
cKOm
FC
FDR
Pyruvate metabolism
Acat1 -1.85 ***
Acot12 -1.51 ***
Acss2 -1.66 ***
Aldh3a2 -1.22 **
Aldh7a1 -1.30 *
Aldh9a1 -1.18 **
Dlat -1.18 *
Fh1 -1.23 *
Grhpr -1.29 *
Ldha -1.45 **
Ldhd -2.68 ***
Mdh1 -1.27 *
Me1 -1.66 ***
Pcx -1.42 ***
Row min (0)
Row max (1)
C
Glutathione metabolism :
NES=-1.746 p-value=0.004 p.adjust=0.042
Running Enrichment Score
Ranked list metric
Rank in Ordered Dataset
Core-enriched genes
Ctrlm
cKOm
FC
FDR
Glutathione metabolism
Chac2 -1.21
Gclm -1.23 *
Ggct -1.85 ***
Ggt1 -2.14 ***
Gpx1 -1.47 **
Gpx4 -1.34 *
Gsr -1.52 ***
Gss -1.35 **
Gstm1 -2.10 ***
Gstm4 -1.25
Gstp1 -1.59 *
Gstp2 -3.49 **
Gstt3 -1.23 *
Idh1 -1.16 0.08
Lap3 -2.03 ***
Ldc1 -5.39 ***
Nat8 -1.63 0.09
Odc1 -2.39 ***
Prdx6 -1.22
Row min (0)
Row max (1)
D
Tryptophan metabolism:
NES=-1.795 p-value=0.004 p.adjust=0.051
Running Enrichment Score
Ranked list metric
Rank in Ordered Dataset
Core-enriched genes
Ctrlm
cKOm
FC
FDR
Tryptophan metabolism
Acat1 -1.85 ***
Acmsd -1.63 *
Aldh3a2 -1.22 **
Aldh7a1 -1.30 *
Aldh8a1 -1.18 *
Aldh9a1 -1.18 **
Aoc1 -1.59 0.09
Cat -1.24 **
Cyp1b1 -1.43 **
Dlst -1.17 0.07
Gcdh -1.52 ***
Haao -1.53 ***
Ido2 -4.17 ***
Inmt -3.54 ***
Kmo -1.70 ***
Kyat1 -1.87 ***
Kyat3 -1.47 **
Tdo2 -9.92 0.06
Row min (0)
Row max (1)

Fig supp. 5

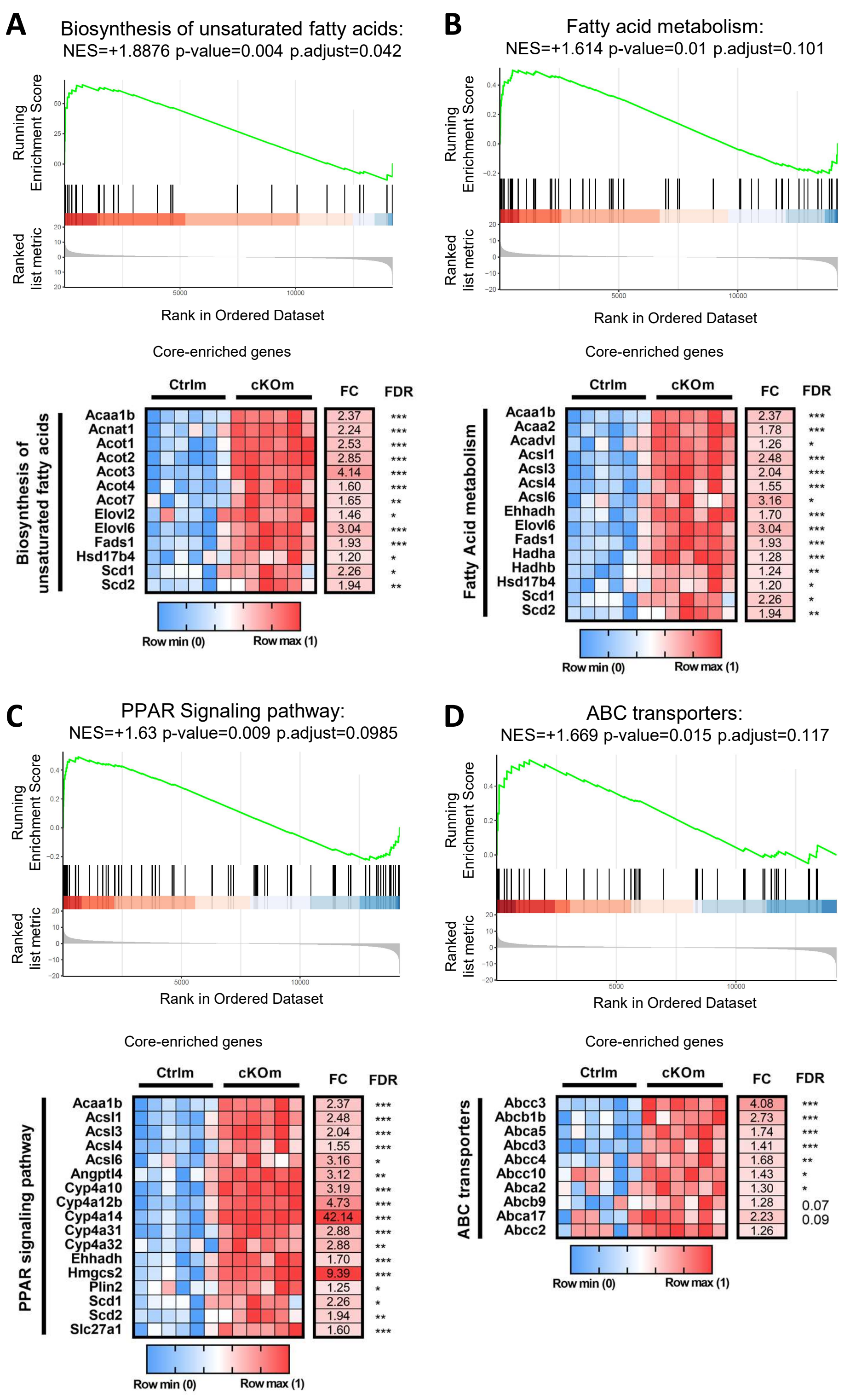

A
Biosynthesis of unsaturated fatty acids:
NES=+1.8876 p-value=0.004 p.adjust=0.042
Running Enrichment Score
Ranked list metric
Rank in Ordered Dataset
Core-enriched genes
Ctrlm
cKOm
FC
FDR
Biosynthesis of unsaturated fatty acids
Acaa1b 2.37 ***
Acnat1 2.24 ***
Acot1 2.53 ***
Acot2 2.85 ***
Acot3 4.14 ***
Acot4 1.60 ***
Acot7 1.65 **
Elovl2 1.46 *
Elovl6 3.04 ***
Fads1 1.93 ***
Hsd17b4 1.20 *
Scd1 2.26 *
Scd2 1.94 **
Row min (0)
Row max (1)
B
Fatty acid metabolism:
NES=+1.614 p-value=0.01 p.adjust=0.101
Running Enrichment Score
Ranked list metric
Rank in Ordered Dataset
Core-enriched genes
Ctrlm
cKOm
FC
FDR
Fatty Acid metabolism
Acaa1b 2.37 ***
Acaa2 1.78 ***
Acadvl 1.26 *
Acsl1 2.48 ***
Acsl3 2.04 ***
Acsl4 1.55 ***
Acsl6 3.16 *
Ehhadh 1.70 ***
Elovl6 3.04 ***
Fads1 1.93 ***
Hadha 1.28 ***
Hadhb 1.24 **
Hsd17b4 1.20 *
Scd1 2.26 *
Scd2 1.94 **
Row min (0)
Row max (1)
C
PPAR Signaling pathway:
NES=+1.63 p-value=0.009 p.adjust=0.0985
Running Enrichment Score
Ranked list metric
Rank in Ordered Dataset
Core-enriched genes
Ctrlm
cKOm
FC
FDR
PPAR signaling pathway
Acaa1b 2.37 ***
Acsl1 2.48 ***
Acsl3 2.04 ***
Acsl4 1.55 ***
Acsl6 3.16 *
Angptl4 3.12 **
Cyp4a10 3.19 ***
Cyp4a12b 4.73 ***
Cyp4a14 42.14 ***
Cyp4a31 2.88 ***
Cyp4a32 2.88 **
Ehhadh 1.70 ***
Hmgcs2 9.39 ***
Plin2 1.25 *
Scd1 2.26 *
Scd2 1.94 **
Slc27a1 1.60 ***
Row min (0)
Row max (1)
D
ABC transporters:
NES=+1.669 p-value=0.015 p.adjust=0.117
Running Enrichment Score
Ranked list metric
Rank in Ordered Dataset
Core-enriched genes
Ctrlm
cKOm
FC
FDR
ABC transporters
Abcc3 4.08 ***
Abcb1b 2.73 ***
Abca5 1.74 ***
Abcd3 1.41 ***
Abcc4 1.68 **
Abcc10 1.43 *
Abca2 1.30 *
Abcb9 1.28 0.07
Abca17 2.23 0.09
Abcc2 1.26
Row min (0)
Row max (1)

Fig supp. 6

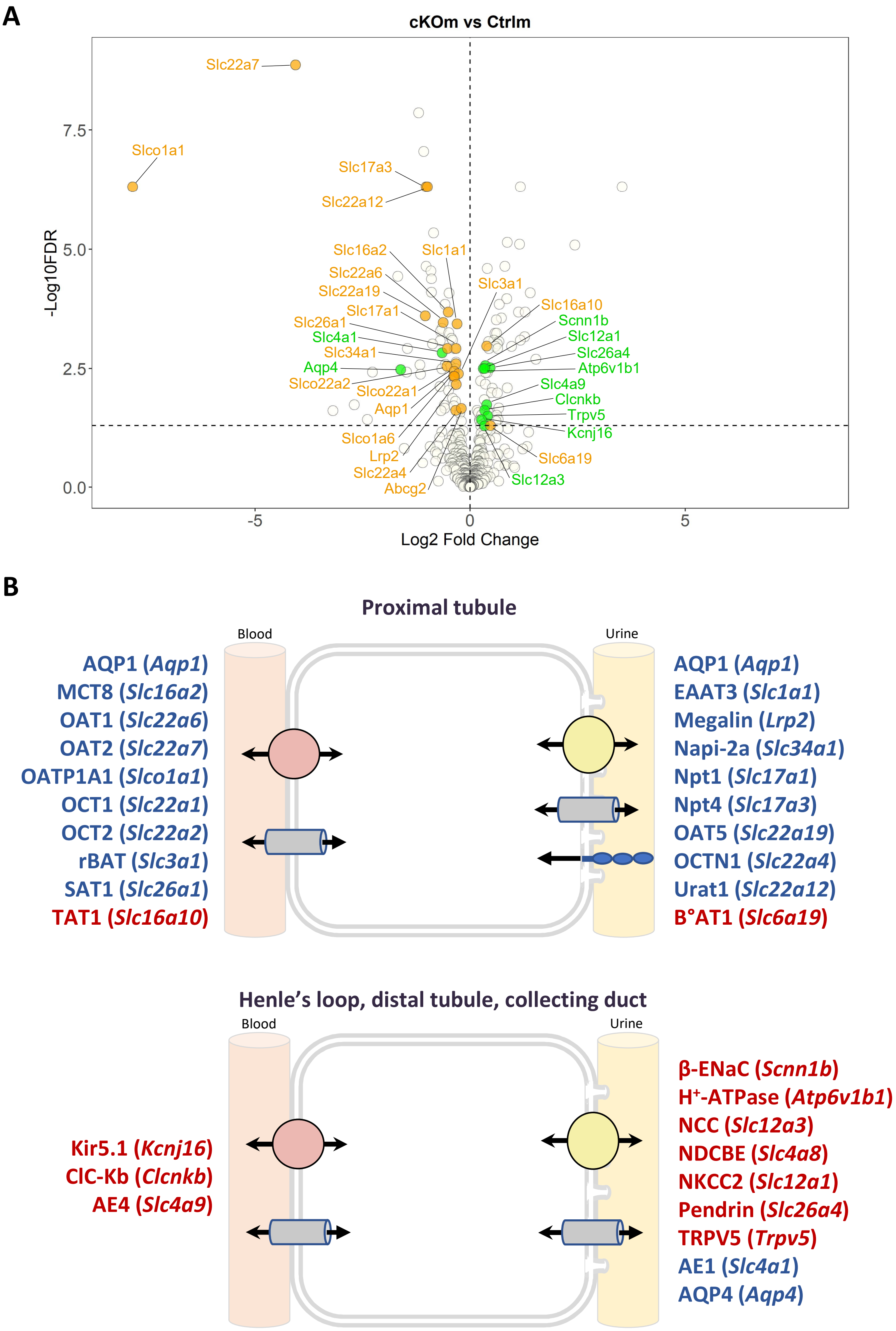
A
cKOm vs Ctrlm
-Log10FDR
Log2 Fold Change
7.5
5.0
2.5
0.0
-5
0
5
Slc22a7
Slco1a1
Slc17a3
Slc22a12
Slc16a2
Slc1a1
Slc22a6
Slc22a19
Slc3a1
Slc17a1
Slc16a10
Slc26a1
Scnn1b
Slc4a1
Slc12a1
Slc34a1
Slc26a4
Aqp4
Atp6v1b1
Slco22a2
Slc22a1
Slc4a9
Aqp1
Clcnkb
Trpv5
Slco1a6
Kcnj16
Lrp2
Slc6a19
Slc22a4
Slc12a3
Abcg2
B
Proximal tubule
Blood
Urine
AQP1 (Aqp1)
MCT8 (Slc16a2)
OAT1 (Slc22a6)
OAT2 (Slc22a7)
OATP1A1 (Slco1a1)
OCT1 (Slc22a1)
OCT2 (Slc22a2)
rBAT (Slc3a1)
SAT1 (Slc26a1)
TAT1 (Slc16a10)
AQP1 (Aqp1)
EAAT3 (Slc1a1)
Megalin (Lrp2)
Napi-2a (Slc34a1)
Npt1 (Slc17a1)
Npt4 (Slc17a3)
OAT5 (Slc22a19)
OCTN1 (Slc22a4)
Urat1 (Slc22a12)
B°AT1 (Slc6a19)
Henle's loop, distal tubule, collecting duct
Blood
Urine
Kir5.1 (Kcnj16)
ClC-Kb (Clcnkb)
AE4 (Slc4a9)
β-ENaC (Scnn1b)
H+-ATPase (Atp6v1b1)
NCC (Slc12a3)
NDCBE (Slc4a8)
NKCC2 (Slc12a1)
Pendrin (Slc26a4)
TRPV5 (Trpv5)
AE1 (Slc4a1)
AQP4 (Aqp4)

Fig supp. 7

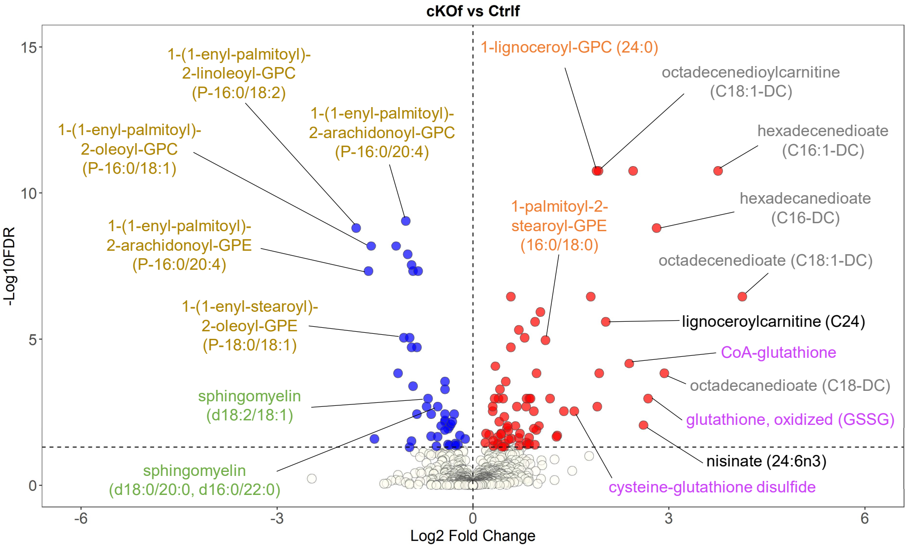

cKOf vs Ctrlf
-Log10FDR
Log2 Fold Change
1-(1-enyl-palmitoyl)-
2-linoleoyl-GPC
(P-16:0/18:2)
1-(1-enyl-palmitoyl)-
2-oleoyl-GPC
(P-16:0/18:1)
1-(1-enyl-palmitoyl)-
2-arachidonoyl-GPC
(P-16:0/20:4)
1-(1-enyl-palmitoyl)-
2-arachidonoyl-GPE
(P-16:0/20:4)
1-(1-enyl-stearoyl)-
2-oleoyl-GPE
(P-18:0/18:1)
sphingomyelin
(d18:2/18:1)
sphingomyelin
(d18:0/20:0, d16:0/22:0)
1-lignoceroyl-GPC (24:0)
octadecenedioylcarnitine
(C18:1-DC)
hexadecenedioate
(C16:1-DC)
hexadecanedioate
(C16-DC)
1-palmitoyl-2-
stearoyl-GPE
(16:0/18:0)
octadecenedioate (C18:1-DC)
lignoceroylcarnitine (C24)
CoA-glutathione
octadecanedioate (C18-DC)
glutathione, oxidized (GSSG)
nisinate (24:6n3)
cysteine-glutathione disulfide

Fig supp. 8

PEX5

ACTIN

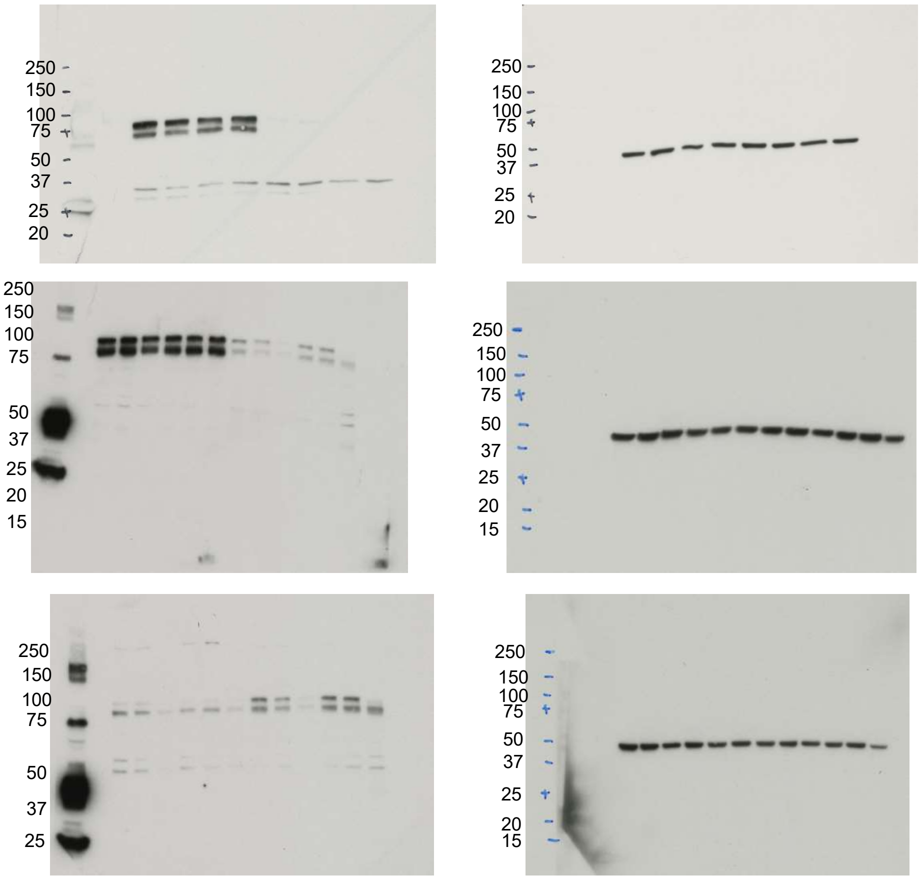

Figure 1B
Upper panel

Figure 1B
Middle panel

Figure 1B
Lower panel

Fig supp. 9
